# Multi-Omics Analysis of Anti-Inflammatory Action of Alkaline Extract of the Leaves of *Sasa* sp.

**DOI:** 10.3390/jcm10102100

**Published:** 2021-05-13

**Authors:** Hiroshi Sakagami, Sachie Nakatani, Ayame Enomoto, Sana Ota, Miku Kaneko, Masahiro Sugimoto, Misaki Horiuchi, Kazuki Toeda, Takaaki Oizumi

**Affiliations:** 1 Meikai University Research Institute of Odontology (M-RIO), Meikai University School of Dentistry, 1-1 Keyakidai, Sakado, Saitama 350-0283, Japan; 2 Graduate School of Pharmaceutical Sciences, Josai University, 1-1 Keyakidai, Sakado, Saitama 350-0295, Japan; s-nakata@josai.ac.jp; 3 Institute for Advanced Biosciences, Keio University, Tsuruoka 997-0052, Japan; ayame.e@ttck.keio.ac.jp (A.E.); sana.ota@ttck.keio.ac.jp (S.O.); KKK-miku@ttck.keio.ac.jp (M.K.); mshrsgmt@tokyo-med.ac.jp (M.S.); 4 Research and Development Center for Minimally Invasive Therapies, Institute of Medical Science, Tokyo Medical University, Shinjuku, Tokyo 160-0022, Japan; 5 Daiwa Biological Research Institute Co., Ltd., Sakado 3-2-1, Takatsu-ku, Kawasaki, Kanagawa 213-0012, Japan; m_horiuchi@daiwaseibutsu.co.jp (M.H.); k_toeda@daiwaseibutsu.co.jp (K.T.); takaakio@daiwaseibutsu.co.jp (T.O.)

**Keywords:** alkaline extract of the leaves of *Sasa* sp., human gingival fibroblast, IL-1β, anti-inflammation, PGE_2_, metabolomics, DNA array, cell survival, apoptosis

## Abstract

Efficient utilization of alkaline extracts of several plants for the treatment of oral diseases has been reported. To investigate the mechanism of anti-inflammatory activity of alkaline extract of the leaves of *Sasa* sp. (SE), multi-omics analysis using metabolomics and DNA array was performed. Human gingival fibroblasts (HGFs) were treated for IL-1β to induce inflammation (detected by PGE_2_ production in culture medium) in the presence or absence of SE. Both IL-1β and SE showed slight hormetic growth stimulation against HGF. SE inhibited PGE_2_ production dose- and time-dependently. Its inhibitory action was more pronounced by first treating the cells with SE, rather than with IL-1β. At 3 h after IL-1β treatment, 18 amino acids (except cysteine and glutamic acid), total glutathione (GSH, GSSG, Cys-GSH disulfide), Met-sulfoxide, 5-oxoproline, and SAM declined, whereas DNA expressions of AKT, CASP3, and CXCL3 were elevated. These changes were reversed by simultaneous treatment with SE. The present study suggests that the anti-inflammatory action of SE is mediated via various metabolic pathways for cell survival, apoptosis, and leukocyte recruitment.

## 1. Introduction

Alkaline extracts of many plants such as licorice root [1], green tea leaf, oolong tea leaf and orange flower [2] have been reported to show much higher anti-HIV activity than water extracts of counterpart plants, possibly due to higher amount of lignin–carbohydrate complexes, which are more efficiently extracted by alkaline solution than hot water. In fact, the anti-HIV activity of the aklaine extract of bamoo leaf declined by removal of the lignin–carbohydrate complex [3]. Although many papers have reported the utilization of alkaline extract for the treatment of oral disease, mechanistic analysis of biological actions such as bone regeneration and anti-inflammation [4], periodontal inflammation and alveolar bone loss [5], anti-oral bacterial activity [6], and alleviation of oral lichenoid dysplasia [7] by alkaline extract has been limited.

Alkaline extract of the leaves of *Sasa* sp. (SE) showed potent anti-viral activity, and its chemotherapeutic index (safety margin) (SI) (determined by the ratio of CC_50_ (50% cytotoxic concentration in uninfected cells)/EC_50_ (50% protective concentration of HIV-infected cells) was 86, comparable with that of lignin–carbohydrate complex (SI = 12~311 (mean: 76) (*n* = 6) and higher than those of hundreds of chemically defined tannins (SI = 1.1~7.3) (*n* = 75) and flavonoids (SI = 1.5~24) (*n* = 114) [8]. Furthermore, SE showed potent anti-inflammation activity (assessed by inhibition of PGE_2_ production and COX-2 protein expression [8] and interleukin (IL)-8 production [6]) against an IL-1β-induced inflammation model with human gingival fibroblasts (HGFs) [9]. Although many researchers have isolated and determined the chemical structures of thousands of low molecular weight compounds, such as tannins and flavonoids, from the natural kingdom, the yield of purified materials is in the milligram order. In contrast, SE can be obtained in the gram order, and is thus suitable for manufacturing over-the-counter (OTC) drugs such as oral rinse and toothpaste [10]. Upon high-performance liquid chromatography (Develosil RPAQUEOUS (C-30), SE was eluted as a single peak at the retention time of 22.175 min, suggesting that SE is present in a multi-biological complex [11]. Among nearly 30 papers on the anti-inflammatory activity of bamboo extracts, only one paper other than our papers used the alkaline extract [12]. All of them focused on the later stage of inflammation, and none of them considered the dental application.

To clarify the action point of SE, optimal condition for induction of inflammation by IL-1β was first investigated. Since inflammation starts as early as 3 h after IL-1β stimulation, the intracellular metabolites and genes that are upregulaed by IL-1β and then downregulated by SE at the earlier stage of inflammation of HGF were identified by metabolomics and DNA microarray analyses, respectively. The earlier stage of inflammation was chosen for the analysis, since at the later stage many different signals may exert non-specific biological actions.

## 2. Experimental Section

### 2.1. Materials

The following chemicals and reagents were purchased from the indicated companies: Dulbecco’s modified Eagle’s medium (DMEM) from GIBCO BRL (Grand Island, NY, USA); fetal bovine serum (FBS) and 3-(4,5-dimethylthiazol-2-yl)-2,5-diphenyltetrazolium bromide (MTT) from Sigma-Aldrich Inc. (St. Louis, MO, USA); dimethyl sulfoxide (DMSO) from Wako Pure Chem. Ind. (Osaka, Japan); 96-microwell plate from TPP (Techno Plastic Products AG, Trasadingen, Switzerland); a 10 cm dish from Becton Dickinson Labware (Franklin Lakes, NJ, USA); interleukin (IL)-1β, R&D systems (Minneapolis, MN, USA).

SE was prepared by iron ion substitution, alkaline extraction, and neutralization/desalting. Lyophilization and measurement of the dry weight of SE showed that it contained 58.2 ± 0.96 mg solid materials/mL [10,13].

### 2.2. Cell Culture

Human gingival fibroblasts (HGFs), human periodontal ligament fibroblasts (HPLFs), and human pulp cells (HPCs) were obtained from the first premolar extracted tooth in the lower jaw and periodontal tissues of a twelve-year-old girl, according to the guideline of the Institutional Board of Meikai University Ethics Committee (No. A0808) after obtaining informed consent from the patients [14]. For the present study, cells at the population doubling level (PDL) of 10–15 were cultured in DMEM supplemented with 10% heat-inactivated FBS, 100 units/mL penicillin G, and 100 μg/mL streptomycin sulfate under humidified 5% CO_2_ atmosphere.

### 2.3. Assay for Cytotoxic Activity

Cells were seeded at 2 × 10^3^ cells/0.1 mL in the inner 60 wells of a 96-microwell plate (Falcon Becton Dickinson, Franklin Lakes, NJ, USA). The surrounding 36 exterior wells were filled with 0.1 mL of sterilized water to minimize the evaporation of water from the culture medium. After 48 h, the medium was removed by suction with an aspirator, and replaced with 0.1 mL of fresh medium containing different concentrations of the sample. Cells were incubated for the indicated times, and the relative viable cell number was then determined by the MTT method [9]. Briefly, the treated cells were incubated for another 2 h in a fresh culture medium containing 0.2 mg/mL MTT. Cells were then lysed with 0.1 mL of DMSO and the absorbance at 560 nm of the cell lysate was determined using a microplate reader (Infinite F 50 R, TECAN, Kawasaki, Japan).

### 2.4. Assay for Pro-Inflammatory Substances

The concentration of PGE_2_ released into the culture medium was determined by enzyme immunoassay (EIA) (Cayman Chemical Co., Ann Arbor, MI, USA).

### 2.5. Processing for Metabolomic Analysis

Near confluent cells in a 10 cm dish were treated for 3 h with 0 (control), IL-1β (3 ng/mL), SE (1%) or IL-1β (3 ng/mL) + SE (1%). Aliquots of the cells were trypsinized and the viable cell number was counted with a hemocytometer after staining with trypan blue. The remaining cells were washed twice with 5 mL of ice-cold 5% D-mannitol and then immersed for 10 min in 1 mL of methanol containing internal standards (25 μM each of methionine sulfone, 2-[*N*-morpholino]-ethanesulfonic acid and D-camphor-10-sulfonic acid). The methanol extract (supernatant) was collected. The aqueous layer was filtered to remove large molecules by centrifugation through a 5 kDa cut-off filter (Millipore, Billerica, MA, USA) at 9100× *g* for 2.5 h at 4 °C. The 320 μL of the filtrate was concentrated by centrifugation and dissolved in 50 μL of Milli-Q water containing reference compounds (200 μM each of 3-aminopyrrolidine and trimesate) immediately before capillary electrophoresis-time-of-flight-mass spectrometry (CE-TOF-MS) analysis. The parameters of the measurement instrument and data processing have already been described. The concentrations of intracellular metabolites were expressed as amol/cell [15,16].

### 2.6. DNA Microarray Processing

Total RNA was extracted from the cells using RNA iso plus reagent (Takara Bio, Tokyo, Japan) according to the manufacturer’s instructions. RNA quantity and quality were determined using the Nano Drop 2000 (Thermo Fisher Scientific K. K., Tokyo, Japan) and RNA 6000 Nano Assay kit on an Agilent 2100 BioAnalyzer (Agilent Technologies Japan, Ltd., Tokyo, Japan), as recommended. RNA samples were used for cRNA target preparation only when the ratio A260:A280 was 1.8–2.1 and the RNA Integrity Number (RIN) value was >7.8.

DNA microarray was performed on the duplicated samples of 4 treatment groups. The Sure-Print G3 Human GE 8 × 60 K Ver. 3.0 array kit (Agilent Technologies Japan, Tokyo, Japan) was used for cRNA target preparation and DNA microarray hybridization detection. Following an assessment of total RNA concentration and quality, 200 ng of the RNA sample from each cell was used for microarray experiments. RNA Spike-In one-color kit (Agilent Technologies Japan, Tokyo, Japan) was used to efficiently monitor their microarray workflow for accuracy. Synthesis of first-strand and second-strand cDNA was performed with T7 promoter primer. Cy3-labeled cRNA was amplified with T7 RNA polymerase Blend and cyanine3-CTP at 40 °C for 2 h and purified using the RNAeasy kit (Qiagen, K.K., Tokyo, Japan). The resulting purified Cy3-labeled cRNA was assessed for concentration, purity, and quantity. In total, 600 ng of Cy3-labeled cRNA was fragmented at 60 °C for 30 min with the fragmentation buffer of Gene Expression Hybridization Kit (Agilent, Tokyo, Japan). The fragmented cRNA with 2× GE Hybridization buffer HI-RPM was loaded into the array slide and hybridized at 65 °C for 17 h. The microarrays were washed with Gene Expression Wash Buffer 1 (Agilent, Tokyo, Japan) at room temperature for 1 min and with Wash Buffer 2 (Agilent, Tokyo, Japan) at 37 °C for 1 min. After drying, the microarrays were immediately scanned with a DNA Microarray Scanner (Agilent, Tokyo, Japan) at 3 μm.

Initial data analysis was performed using Feature Extraction software (Agilent, Tokyo, Japan) to exclude outlier pixels, accurately determine feature intensities and ratios, and calculate statistical confidences. Raw intensities were normalized using a 75th percentile global normalization and law signal cut-off for each spot before further analysis of the quantified data using Subio Platform Basic Plug-in (Subio Inc., Aichi, Japan).

Further information concerning gene function, functional annotation clustering, and the biological and molecular function of each gene analyzed was obtained with Subio Platform Advanced Plug-in (Subio Inc., Aichi, Japan) and KeyMolnet Lite (IMMD Inc., Tokyo, Japan) software.

### 2.7. Statistical Analysis

Statistical analyses were performed using the Origin Pro 2018 software (Origin Lab Corporation, MA, USA). Experimental data are presented as the mean ± standard deviation (SD) of triplicate determinations. The statistical analysis was performed using one-way analysis of variance (ANOVA) followed by Bonferroni’s post hoc test for multiple comparisons. A value of *p* < 0.05 was considered to indicate statistically significant differences.

## 3. Results

### 3.1. Pretreatment, Rather Than Post-Treatment, with SE More Efficiently Inhibited IL-1β-Stimulated PGE_2_ Production in HGF.t

#### 3.1.1. Optimal Conditions for Induction of Inflammation by IL-1β

**Figure 1:**
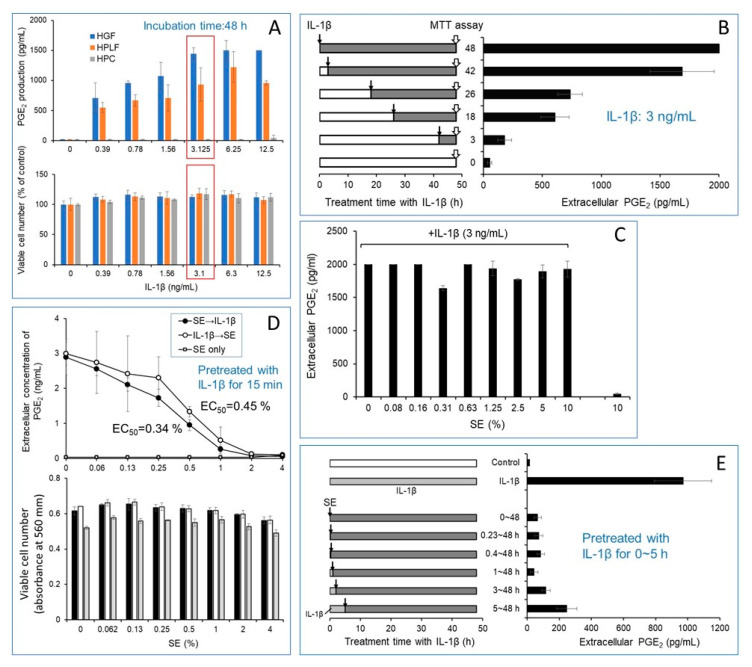
Kinetics of inhibition of IL-1β-induced human gingival fibroblast PGE_2_ production by SE. (**A**) Dose–response: IL-β stimulated the production of PGE_2_ in HGF, HPLF, but not in HPC. (**B**) Time-course of PGE_2_ production after IL-1β (3 mg/mL) addition. (**C**) SE did not interfere with the PGE_2_ measurement. (**D**) SE inhibited the IL-1β-stimulated PGE_2_ production more effectively when HGF cells were first treated by SE before IL-1β addition. (**E**) Prolonged pretreatment with IL-1β (3 mg/mL) reduced the inhibitory effect of SE to a greater extent.

We first set up the optimal condition for measuring the anti-inflammatory action of SE (Figure 1). Interleukin (IL)-1β induced an approximately 100-fold increase in PGE_2_ production in human gingival fibroblasts (HGFs) and human periodontal fibroblasts (HPLFs), but not in human pulp cells (HPCs) (A). The optimal concentration of IL-1β was 3.1~12.5 (ng/mL). Since higher PGE_2_ production was observed in HGF than HPLF, we used HGF in the following experiments. The production of PGE_2_ was observed 3 h after IL-1β (3 ng/mL) treatment and increased up to 48 h (B). The addition of SE up to 10% did not interfere with the actual determination of PGE_2_ with ELISA (C). Unless otherwise stated, cells were treated for 48 h with IL-1β (3 ng/mL).

#### 3.1.2. Inhibition of IL-1β-Induced Inflammation by SE

SE dose-dependently reduced the IL-1β-induced PGE_2_ production. In the presence of 1 or 2% SE, PGE_2_ production was reduced to 9.1 and 3.0% of control, respectively. The inhibitory effect of SE was more pronounced when SE was added before IL-1β addition (D). When HGF cells were preincubated for longer times with IL-1β before the addition of SE, the inhibitory effect of SE was progressively reduced (E). However, approximately 25% of PGE_2_ production remained even if HGF cells were incubated for 5 h with 2% SE (E). This suggests that some parts of SE may bind to IL-1β extracellularly and block its binding to a cellular receptor and then inhibit the signal transduction of IL-1β.

#### 3.1.3. Mild Growth Stimulation Effect of IL-1β and SE

A careful inspection revealed that both IL-1β and SE slightly stimulated the growth of HGF (A, D). Reproduced results were obtained by repeated experiments. This is consistent with our finding that SE stimulates the growth of differentiated rat PC12 neuronal cells in a serum-free medium [17].

### 3.2. Metabolome Analysis

**Figure 2:**
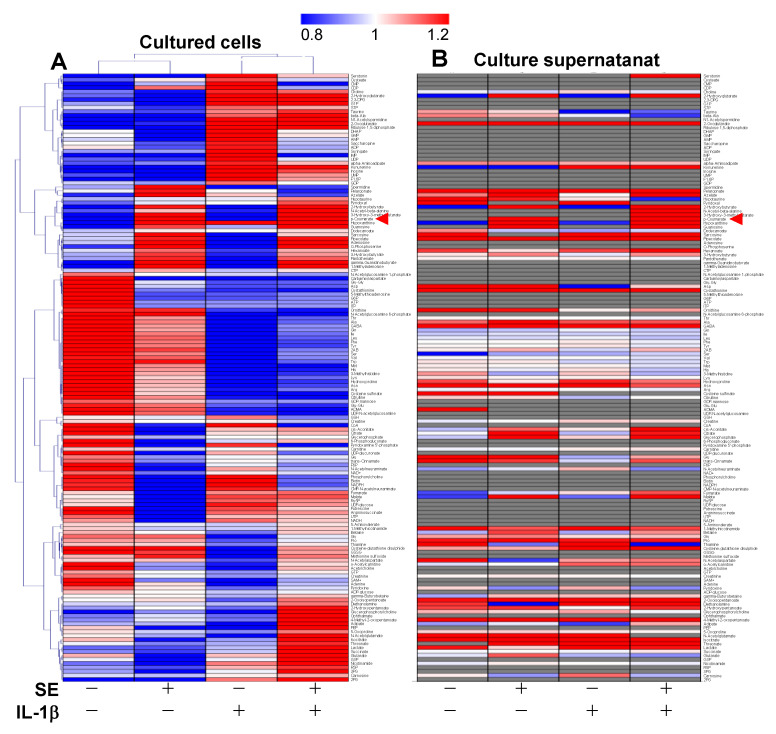
Metabolomic analysis of HGF cells incubated for 3 h without (control) (column 1) or with SE (2%) (column 2), IL-1β (3 ng/mL) (column 3) and SE (2%) + IL-1β (3 ng/mL) (column 4). The averaged concentrations of samples (*n* = 4) for each condition are visualized. To determine the color, the average concentration was calculated for each metabolite, and individual metabolite concentration was divided by the average. The metabolites of the cultured cell (**A**) were aligned by the clustering with Pearson Correlation, and the metabolites of the supernatant (**B**) were aligned according to the data in (**A**). The numbers of cells recovered per dish are 1.06 ± 0.086 × 10^6^, 1.16 ± 0.068 × 10^6^, 1.21 ± 0.12 × 10^6^, and 1.12 ± 0.053 × 10^6^, respectively. Blue, white, and red indicate fold changes of 0.8, 1, and 1.2, respectively, compared to the average of each metabolite. The gray indicates that the corresponding metabolite is not detected. The names of metabolites can be visible by 4-fold magnification with zooming. Original data are available in Supplemental Table S1.

A total of 153 metabolites were detected. *p*-Coumaric acid, one of the precursors of the lignin–carbohydrate complex (LCC) [18,19], was detected in both cells and medium, indicating that SE contains LCC as major anti-viral and anti-inflammatory substances. There were many metabolites whose expressions were increased or decreased by IL1-β or by SE. Interestingly, when column 2 and column 3 were compared (Figure 2A), their patterns of ups and downs were found to not be overlapped with each other.

Treatment of HGF cells with IL-1β resulted in a 38% decline of a total of 18 amino acids (except cysteine and glutamic acid) (Table 1). Cysteine was not detected due to the degradation during sample preparation. Additionally, total glutathione (GSH, GSSG, and cysteine-glutathione disulfide), Met-sulfoxide, 5-oxoproline, and *S*-adenosylmethionine (SAM) declined. These changes were reversed by simultaneous treatment with SE. ATP utilization (assessed by the ratio of AMP/ATP and ADP/ATP) and GTP utilization (assessed by the ratio of GMP/GTP and GDP/GTP) were elevated by 60–88% and 35–44%, respectively, due to IL-1β and were reversed to near the control level by SE (Table 1).

### 3.3. DNA Array Analysis

**Figure 3:**
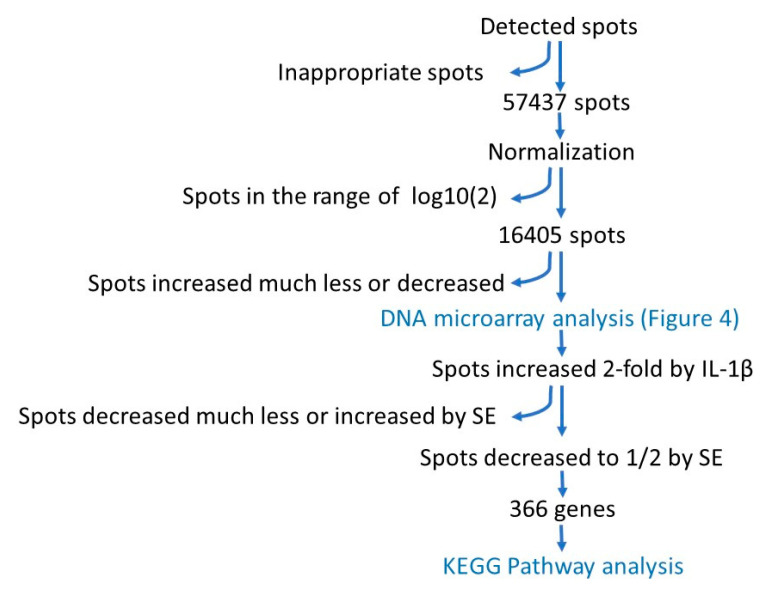
Flow chart of DNA array analysis.

The flow chart of DNA array analysis is shown in Figure 3. After removing inappropriate spots, 57,432 spots were selected. After normalization, the spots within the range of log 2 (2), compared with control, were removed, and the remaining 16,405 spots were subjected to DNA microarray analysis. Next, we extracted the genes whose expressions were enhanced more than twice by IL-1β and reduced to 1/2 by simultaneous addition by SE to obtain 366 genes. These genes were subjected to assay for the KEGG pathway.

**Figure 4:**
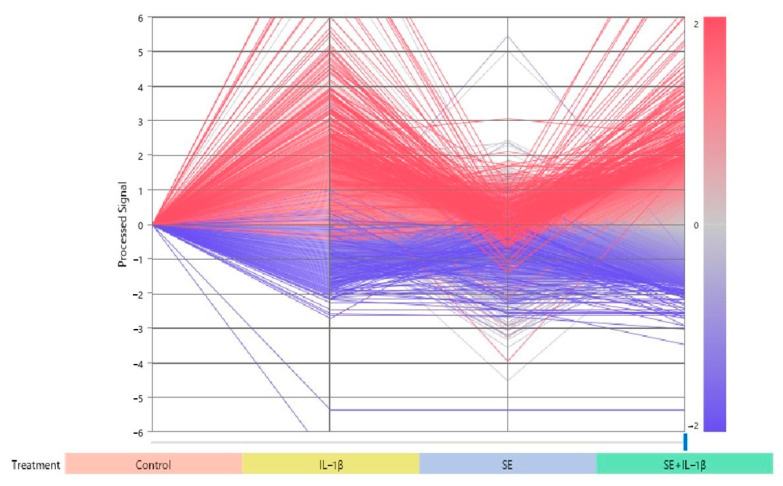
The expression intensity of each sample was expressed in a logarithmic scale using a line graph as a reference for control. In total, 16,405 spots with fluctuations of Log 2 (2) or more compared to the control were analyzed. Genes with higher mean expression intensity compared to the control are shown in red, and genes with lower mean expression intensity are shown in blue.

Changes in the expression intensity of 16,405 genes are shown in Figure 4. There were many genes whose expressions were increased by IL1-β compared to the control, and there was a group of genes in which the increase by IL1-β was suppressed by SE + IL1-β (Figure 4).

**Figure 5:**
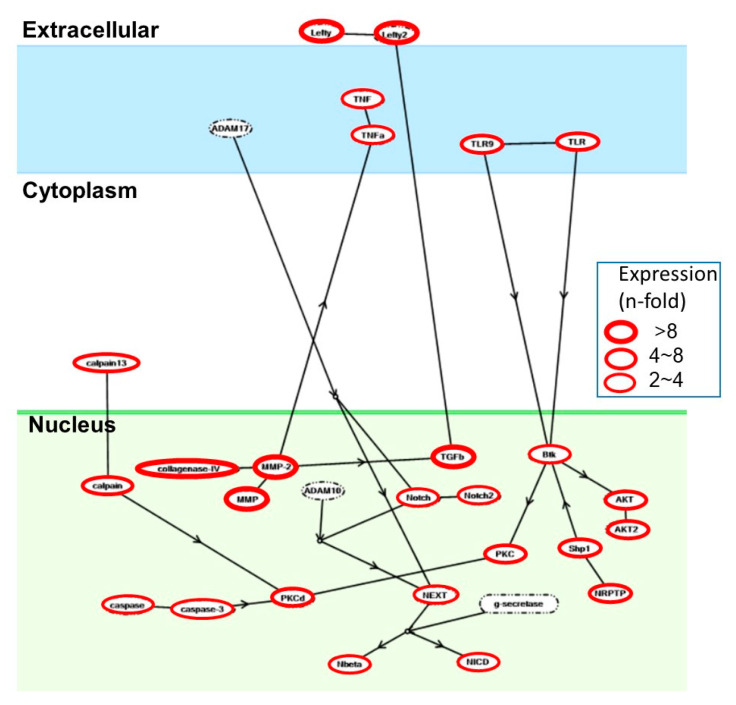
Enhanced mRNA expression of tumor necrosis factor superfamily, member 2 (TNFA), RAC serine/threonine-protein kinase (AKT), caspase 3 (CASP3), and CXC motif chemokine (CXCl13) on the TNF pathway by IL1-β.

IL1-β induced mRNA expression of 26 genes including tumor necrosis factor superfamily member 2 (TNFA) (2^1.439^-fold), RAC serine/threonine-protein kinase (AKT) (2^2.32^-fold), caspase 3 (CASP3) (2^1.027^-fold). These expression levels were suppressed by adding SE at the same time. In particular, CASP3 was induced about twice as much as the control (2^1.072^-fold) by adding IL1-β, but it was returned to the control level by adding SE at the same time (Table 2). On the other hand, chemokines such as CXC motif chemokine on the TNF pathway (CXCL3) were increased to a greater extent (2^8.826^-fold), but their expression did not return to the control level. Highly expressed mRNAs are depicted in Figure 5, where magnitudes of amplification are indicated by the thickness of red circles.

## 4. Discussion

The present study demonstrated that SE inhibited the IL1-β-induced inflammation (PGE_2_ production and other inflammatory cytokines) [9], and the anti-inflammatory action of SE was partially inhibited when HGF cells were treated with SE after pretreatment with IL1-β (Figure 1). This suggests that one of the anti-inflammatory actions of SE may be mediated by the inhibition of IL1-β binding to its cellular receptor (IL1-βR) by masking the ligand or receptor. Since HGF expresses various cell surface receptors of various inflammatory cytokines upon stimulation [17], SE may non-specifically inhibit the ligand–receptor interactions of all cytokines. Further studies are necessary to test this possibility.

SE contains numerous components including the lignin–carbohydrate complex and its degradation products [13]. Such components bind with each other, producing the aggregated form [11]. At present, it is not clear which components or aggregate formations are responsible for the anti-inflammatory action of SE. The present metabolomic analysis demonstrated that (i) SE contains *p*-coumaric acid, one of the degradation products of lignin–carbohydrate complex, (ii) it was incorporated into the cells (332 atom/cell), and (iii) IL1-β treatment slightly increased its intracellular concentration (511 amol/cell) with the reduction in the extracellular concentration from 146 to 139 µM (Supplementary Table S4). This suggests the possibility that the anti-inflammatory activity of SE might be in part to *p*-CA. However, this possibility was eliminated by our finding that the anti-inflammatory activity of *p*-coumaric acid against HPLF (assessed by chemotherapeutic index CI (=CC_50_/IC_50_)) was much less than SE (chemotherapeutic index >3.1 vs. >96.3) (Supplementary Figure S1). We also found that the anti-inflammatory activity of other popular polyphenols such as curcumin, gallic acid, and ferulic acid was very low (CI = 0.94~2.9). On the other hand, Japanese traditional medicines (Kampo), such as Hangeshashinto [20,21] inhibited IL-1β-stimulated PGE_2_ production, to the comparable extent with that of SE (CI = 100). Endotoxin contamination of Hangeshashinto and SE was 8.7 [22] and <2 ng/g (under the detection limit, possibly due to the cleavage of the ester bond of LPS during alkaline extraction), respectively. These data indicate that the anti-inflammatory activity of SE is higher than popular low molecular weight polyphenols and comparable with that of Kampo medicines.

The present study demonstrated that IL1-β reduced approximately 40% of intracellular concentrations of amino acids, which was reversed to some extent by the addition of SE (Table 3). At present, the biological significance of this finding is not clear. Similarly, SE rescued the IL1-β-induced decline of total glutathione and Met-sulfoxide and 5-oxoproline to some extent. This is consistent with our previous finding that Met-sulfoxide and 5-oxoproline declined during the aggravation of IL1-β-induced HGF inflammation by TiO_2_ nanoparticles (Supplementary Figure S2A,B, cited from [15]). Since Met-sulfoxide [23,24,25] and 5-oxoproline [26,27,28] are well-known markers reflecting oxidative stress, SE might have induced the cells to fight against oxidative stress.

We have previously reported that SAM level step-wisely declined with aggravation of inflammation triggered by IL1-β then TiO_2_ nanoparticles (Supplementary Figure S2C). The present study showed that, during recovery from inflammation by SE, SAM returned to near the control level (Table 2). SAM is synthesized in a cyclic pathway (termed one-carbon metabolism) from methionine and ATP by methionine adenosyltransferase (EC 2.5.1.6). During inflammation, methionine and ATP levels decline, thus leading to the decline of SAM, which is a well-known methyl group transfer. Since methylation of DNA and histone is known to repress the expression of various genes, a poor supply of SAM may lead to the enhanced expression of pro-inflammatory cytokines [29]. Therefore, reversal of SAM by SE can be considered to be the trigger of anti-inflammatory action.

IL-1β is an important mediator of the inflammatory response and is involved in a variety of cellular activities, including cell proliferation, differentiation, and apoptosis. The present DNA microarray analysis demonstrated the activation of four independent pathways by IL1-β, which were also reversed by SE: tumor necrosis factor superfamily member 2 (TNFA), RAC serine/threonine-protein kinase (AKT), and caspase 3 (CASP3) (Figure 5, Table 3). An increase in the ATP and GTP utilization favors the commitment of cells towards apoptosis since the elevation of cytosolic ATP level is a requisite to the apoptotic cell death process [30]. Several papers have reported the mild apoptosis induction (detected by annexin staining) of HGF by IL-1β [31]. IL-1β also induced mild growth stimulation of HGF by upregulation of NF-κB pathway members [32], supporting our data. IL-1β also increased matrix metalloproteinase (MMP) levels [33,34]. Simultaneous stimulation with transforming growth factor (TGF)-β1 or IL-1β induce weak apoptosis and then augment IL-8 and VEGF production [35]. Such weak apoptosis may induce proinflammatory cytokines and adherent molecules by HGF, leading to the exacerbation of periodontal disease.

IL-1β induces the activation of Akt by 473 serine phosphorylation, in a PI-3K-dependent manner, and the inhibition of Akt prevents IL-1β-mediated differential embryo-chondrocyte expressed gene 1 (DEC1) and HIF-1α induction in HGF. Although IL-1β induced the expression chemokine CXCL10 and attached factor ICAM-1, in synergy with oncostatin M [36], the inhibition of CXCL10 by SE was mild (Table 2). This suggests that chemokines may not be tightly involved in the anti-inflammatory action of SE.

We have previously shown that SE and lignin–carbohydrate, both extracted by alkaline solution, showed potent anti-viral activity. Their anti-viral potential [37,38,39,40,41,42,43] is two-fold higher than that of tannins and flavonoids [44,45,46]. Recently, SE instantly inactivated both HSV and HIV, and their chemotherapeutic index is much higher than povidone-iodine. This suggests the superiority of SE over povidone-iodine for mouth wash for preventing virus infection [10]. A combination of presently described anti-inflammatory activity and potent anti-viral activity may synergistically improve the oral environment.

In conclusion, the present study demonstrated, for the first time, that the anti-inflammatory action of SE is mediated via various metabolic pathways for cell survival, apoptosis, and leukocyte recruitment. Since SE showed potent anti-inflammatory activity, in addition to its potent anti-viral activity reported previously, the applicability of SE to oral inflammation and virally induced oral diseases was suggested.

## Figures and Tables

**Table 1:** Metabolites downregulated by IL-1β and reversed by SE.

	Metabolites (Amol/Cell)							
	Control	(%)	IL-1β	(%)	SE	(%)	SE + IL-1β	(%)
**Amino acids**								
Gly	211,177	100	146,168	69	211,841	100	185,667	88
Ala	60,888	100	33,272	55	51,251	84	40,898	67
Arg	9902	100	6464	65	8236	83	7077	71
Asp	26,445	100	17,409	66	19,456	74	17,654	67
Asn	15,192	100	8635	57	11,970	79	9718	64
Gln	337,154	100	197,771	59	283,383	84	241,164	72
Glu	287,270	100	258,709	90	239,064	83	255,679	89
His	13,301	100	7407	56	10,625	80	8041	60
Ile	35,810	100	22,531	63	32,087	90	25,843	72
Leu	37,506	100	22,611	60	33,730	90	26,286	70
Lys	24,974	100	15,917	64	20,350	81	17,365	70
Met	15,489	100	7274	47	11,717	76	8548	55
Phe	29,015	100	16,943	58	24,954	86	19,252	66
Pro	62,148	100	49,904	80	61,787	99	56,773	91
Ser	47,587	100	29,061	61	42,219	89	35,215	74
Thr	140,117	100	73,931	53	113,094	81	91,622	65
Trp	6312	100	3447	55	6001	95	4418	70
Tyr	30,049	100	17,177	57	25,465	85	19,564	65
Val	29,763	100	19,547	66	28,292	95	22,768	76
Total	1,420,101	100	954,178	62	1235,525	86	1093,552	71
**Glutathiones**								
GSH	41,743	100	45,315	109	40,377	97	36,927	88
GSSG	19,334	100	6905	36	17,109	88	15,965	83
Cys-GSH disulfide	461	100	27	6	394	85	329	71
Total	61,538	100	52,248	85	57,880	94	53,221	86
**Others**								
Met-sulfoxide	283	100	175	41	316	74	297	149
5-Oxoproline	8806	100	7801	89	8281	94	9507	108
SAM+	713	100	324	45	426	60	443	62
**ATP/GTP utilization**								
ATP	64,581	100	46,778	72	46,906	73	46,787	72
ADP	4074	100	4734	116	3456	85	3729	92
AMP	425	100	581	136	333	78	423	99
AMP/ATP	0.0066	100	0.0124	188	0.0071	108	0.0090	137
ADP/ATP	0.0631	100	0.1012	160	0.0737	117	0.0797	126
GTP	14,959	100	12,730	85	13,880	93	14,106	94
GDP	747	100	861	115	713	95	776	104
GMP	162	100	198	123	123	76	165	102
GMP/GTP	0.0108	100	0.0156	144	0.0089	82	0.0117	109
GDP/GTP	0.0499	100	0.0676	135	0.0514	103	0.0550	110

Each value is the mean of 4 determinations. SD values of each metabolite are available in Supplemental Table S2.

**Table 2:** Metabolites downregulated by IL-1β and reversed by SE.

	Expression (2n-Fold Increase)			
	Control	SE	IL-1β	SE + IL-1β
Btk	0.000	0.000	1.108	0.000
calpain	0.000	0.000	2.139	0.000
calpain 13	0.000	0.000	2.139	0.000
collagenase-IV	0.000	0.000	3.232	0.000
Lefty	0.000	0.000	3.521	0.000
Lefty2	0.000	0.000	3.521	0.000
MMP	0.000	0.000	3.232	0.000
MMP-2	0.000	0.000	3.232	0.000
Nbeta	0.000	0.000	1.121	0.000
NEXT	0.000	0.000	1.121	0.000
NICD	0.000	0.000	1.121	0.000
Notch	0.000	0.000	1.121	0.000
Notch2	0.000	0.000	1.121	0.000
NRPTP	0.000	0.000	2.017	0.000
PKC	0.000	0.000	1.676	0.000
PKCd	0.000	0.000	1.676	0.000
Shp1	0.000	0.000	2.017	0.000
TGFb	0.000	0.000	3.521	0.000
TLR	0.000	0.000	2.478	0.000
TLR9	0.000	0.000	2.478	0.000
TNF	0.000	0.000	1.439	0.255
TNFa	0.000	0.000	1.439	0.255
AKT	0.000	−0.189	2.320	−0.309
AKT2	0.000	−0.189	2.320	−0.309
caspase	0.000	−0.705	1.072	−0.151
caspase-3	0.000	−0.705	1.072	−0.151
CXCL3	0.000	0.110	8.826	7.756
CXCL5	0.000	−0.301	3.038	1.990
CXCL10	0.000	0.000	3.254	2.252
CCL7	0.000	0.000	4.148	3.141

Data for a total of 366 gene expressions are available in Supplementary Table S3.

**Table 3:** Summary of anti-inflammatory action of SE against IL-1β-treated HGF cells.

	+ IL-1β		+SE+ IL-1β
**Metabolomic analysis**			
19 Amino acids (except Cys and Glu)	↓	→	↑
Total glutathione (GSH, GSSG, Cys-GSH disulfide)	↓	→	↑
Met-sulfoxide	↓	→	↑
5-Oxoproline	↓	→	↑
SAM	↓	→	↑
**DNA array analysis**			
AKT (Cell survival)	↑	→	↓
CASP3 (Apoptosis)	↑	→	↓
CXCL3 (Leukocyte recruitment)	↑	→	↓

## Data Availability

We have provided our previous data that support our present findings in the supplementary materials Figure S2.

## Supplementary Materials

The following are available online at https://www.mdpi.com/article/10.3390/jcm10102100/s1, Figure S1: Anti-inflammatory activity of p-coumaric acid and other lower molecular polyphenols, Figure S2: Changes in the intracellular concentrations of methionine sulfoxide, 5-oxoproline and SAM after addition of IL-1β (3 ng/mL) and TiO_2_ nanoparticles in HGF. Table S1: Original data for Figure 2, Table S2: Original data with SD values for Table 1, Table S3: Original data for Table 2, Table S4: Intracellular and extracellular concentration of p-coumaric acid.
