# Supplementary material for: Multi-Omics Analysis of Anti-Inflammatory Action of Alkaline Extract of the Leaves of Sasa sp
Source: J Clin Med. 2021 May 13;10(10):2100. doi: 10.3390/jcm10102100 (PMC8152990; doi:10.3390/jcm10102100)
Supplement: Supplementary file 1 [file jcm-10-02100-s001.zip › jcm-1158973-supplementary.pdf]

# Supplementary Figure S1

Anti-inflammatory activity of *p*-coumaric acid and other lower molecular polyphenols

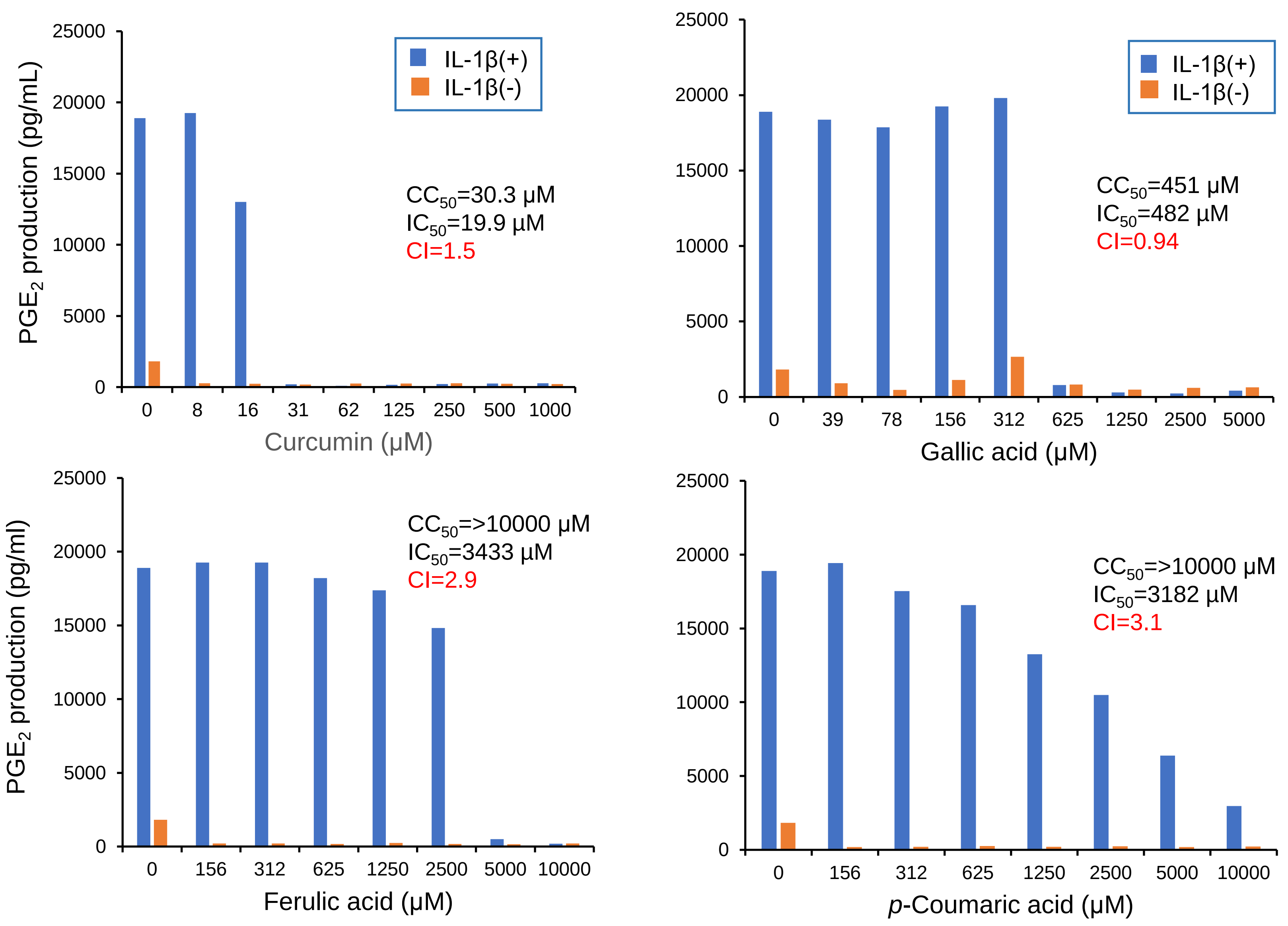

# Supplementary Figure S2

Changes in the intracellular concentrations of methionine sulfoxide, 5-oxoproline and SAM after addition of IL-1β (3 ng/mL) and $TiO_2$ nanoparticles in HGF.

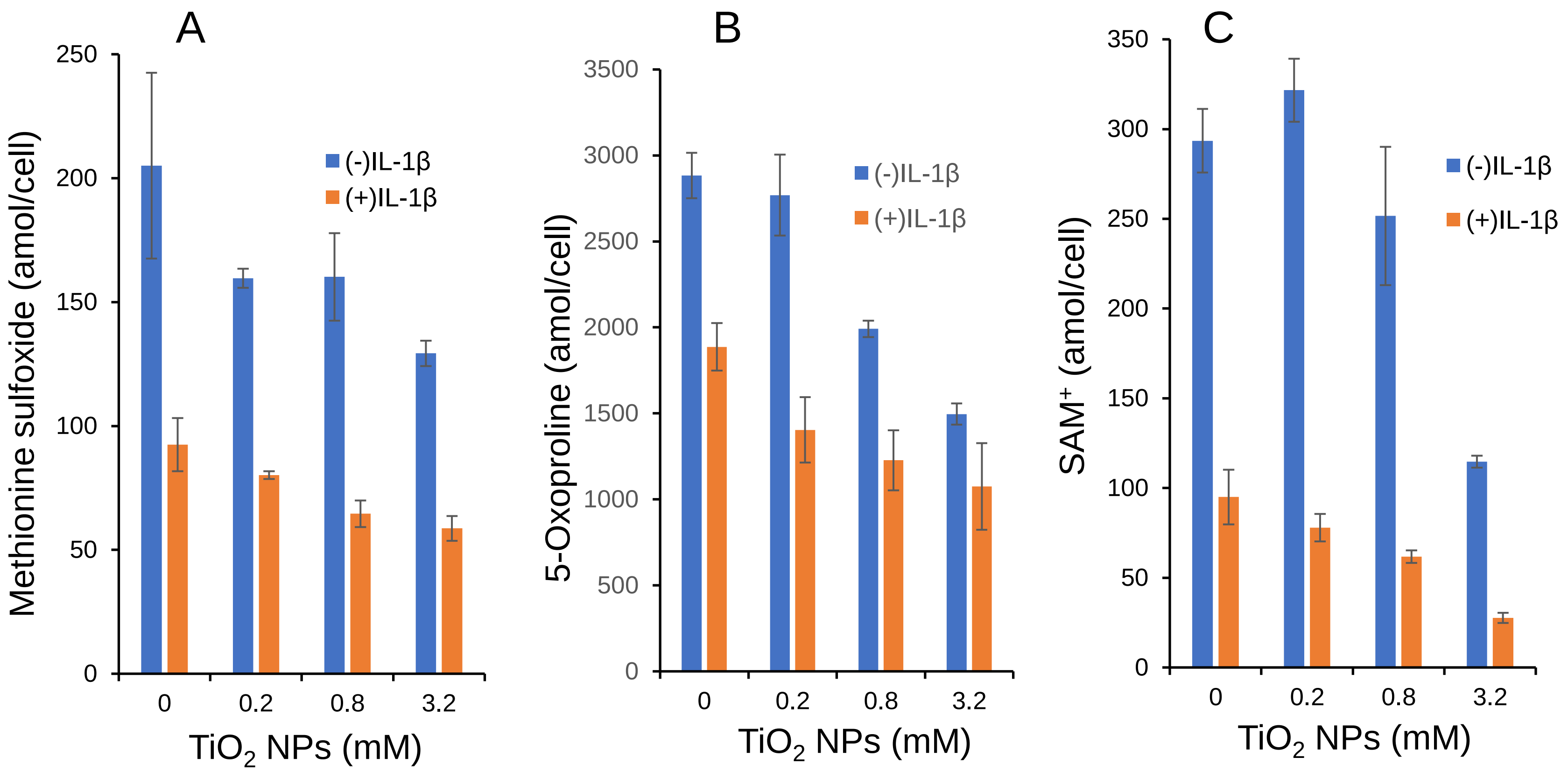


Data from reference our previous paper (Biomaterials 57, 33-40, 2015). Each value represents mean ±S.D. (n=4). 24 h after treatment.

# Supplementary Table S1

# Original data for Figure 2

| mode | Compound Name | Ave m/z | Ave CorMT | 培養細胞 | | | | | | | | | | | | | | | | 培養上清 | | | | | | | | | | | | | | | | | | | | KEGG ID(other) | |
|---|---|---|---|---|---|---|---|---|---|---|---|---|---|---|---|---|---|---|---|---|---|---|---|---|---|---|---|---|---|---|---|---|---|---|---|---|---|---|---|---|---|
| | | | | 細胞1個当たりのmol数(amol/cell) | | | | | | | | | | | | | | | | サンプル中の濃度(μM) | | | | | | | | | | | | | | | | | | | | | |
| | | | | ササヘルス+IL-1β | | | | ササヘルス | | | | IL-1β | | | | コントロール | | | | 地中の試料添加直前のサン | | | | ササヘルス+IL-1β | | | | ササヘルス | | | | IL-1β | | | | コントロール | | | | | |
| | | | | 1.12 ±0.053× $10^6$個の細胞メタノール抽出液 | | | | 1.16±0.058× $10^6$ 個の細胞メタノール抽出液 | | | | 1.21±0.12× $10^6$ 個の細胞メタノール抽出液 | | | | 1.08 ±0.086× $10^6$ 個の細胞メタノール抽出液 | | | | | | | | | | | | | | | | | | | | | | | | | |
| | | | | 1 | 2 | 3 | 4 | 5 | 6 | 7 | 8 | 9 | 10 | 11 | 12 | 13 | 14 | 15 | 16 | 1 | 2 | 3 | 4 | 1 | 2 | 3 | 4 | 5 | 6 | 7 | 8 | 9 | 10 | 11 | 12 | 13 | 14 | 15 | 16 | | |
| Cation | Gly | 76.0412 | 10.47 | 189467 | 179374 | 182203 | 191625 | 217384 | 220154 | 205090 | 204736 | 145414 | 144981 | 146254 | 148021 | 219541 | 201132 | 197780 | 226257 | 401 | 443 | 424 | 445 | 574 | 591 | 595 | 614 | 623 | 641 | 612 | 622 | 578 | 592 | 575 | 584 | 639 | 606 | 577 | 614 | | |
| Cation | Putrescine(1,4-Butanediamine) | 89.1085 | 6.31 | 204 | 93 | 237 | 268 | 138 | 218 | 123 | 0 | 216 | 161 | 239 | 270 | 248 | 216 | 256 | 209 | 0 | 0 | 0 | 0 | 0 | 0 | 0 | 0 | 0 | 0 | 0 | 0 | 0 | 0 | 0 | 0 | 0 | 0 | 0 | 0 | | |
| Cation | Sarcosine | 90.0576 | 11.72 | 182 | 264 | 175 | 229 | 271 | 236 | 173 | 378 | 188 | 200 | 191 | 0 | 314 | 0 | 253 | 221 | 2.7 | 0 | 0 | 2.0 | 2.5 | 0 | 0 | 0 | 0 | 0 | 0 | 0 | 0 | 0 | 0 | 3.0 | 0 | 2.5 | 0 | 0 | | |
| Cation | beta-Ala | 90.0573 | 9.41 | 9455 | 8870 | 8973 | 9591 | 5717 | 5732 | 5345 | 5382 | 7935 | 7612 | 7726 | 8087 | 5421 | 5462 | 4886 | 5360 | 3.9 | 4.5 | 4.3 | 5.0 | 3.3 | 3.3 | 4.0 | 4.2 | 5.0 | 4.4 | 4.5 | 4.8 | 4.8 | 3.9 | 4.3 | 4.4 | 5.1 | 4.2 | 4.6 | 5.4 | | |
| Cation | Ala | 90.0573 | 11.26 | 41829 | 40514 | 40557 | 40692 | 51987 | 52675 | 50337 | 50006 | 33922 | 32978 | 33492 | 32695 | 64847 | 56747 | 57789 | 64170 | 95 | 103 | 100 | 98 | 299 | 307 | 310 | 333 | 337 | 348 | 330 | 352 | 303 | 329 | 304 | 318 | 331 | 305 | 292 | 305 | C00133 | |
| Cation | 2AB | 104.0723 | 11.91 | 894 | 948 | 887 | 939 | 1194 | 1239 | 1157 | 1130 | 661 | 670 | 670 | 663 | 1365 | 1308 | 1263 | 1363 | 7.9 | 6.9 | 7.6 | 7.7 | 8.5 | 8.6 | 9.3 | 9.0 | 8.6 | 9.2 | 8.6 | 9.1 | 8.0 | 8.1 | 8.2 | 8.1 | 8.3 | 8.7 | 8.2 | 8.8 | C02261 | |
| Cation | GABA | 104.0725 | 9.81 | 632 | 696 | 687 | 735 | 800 | 871 | 803 | 758 | 591 | 550 | 546 | 552 | 950 | 1033 | 874 | 927 | 0 | 0 | 0 | 0 | 1.0 | 1.2 | 1.1 | 1.2 | 1.3 | 1.2 | 1.1 | 1.3 | 0 | 0 | 0.77 | 0 | 0.73 | 0 | 0 | 0.84 | | |
| Cation | Choline | 104.1092 | 8.87 | 5691 | 5198 | 5540 | 6043 | 3918 | 4361 | 3957 | 3972 | 6011 | 5960 | 5640 | 5853 | 4462 | 4337 | 4439 | 4661 | 60 | 61 | 57 | 60 | 59 | 59 | 57 | 52 | 58 | 57 | 56 | 52 | 61 | 62 | 59 | 56 | 57 | 58 | 58 | 55 | | |
| Cation | Ser | 106.0520 | 12.23 | 35614 | 35384 | 33765 | 36099 | 42278 | 42914 | 43911 | 39775 | 31109 | 28504 | 28588 | 28042 | 49233 | 47842 | 45096 | 48177 | 389 | 404 | 398 | 380 | 274 | 278 | 277 | 272 | 273 | 266 | 270 | 252 | 272 | 271 | 264 | 261 | 224 | 229 | 220 | 235 | C00740 | C00716 |
| Cation | Diethanolamine | 106.0879 | 9.77 | 2311 | 6060 | 1244 | 7242 | 7840 | 1353 | 874 | 4807 | 5707 | 817 | 6420 | 1489 | 4733 | 1044 | 6240 | 2711 | 5.6 | 4.8 | 11 | 6.4 | 36 | 9.4 | 5.9 | 9.7 | 4.1 | 11 | 5.5 | 4.0 | 11 | 6.6 | 9.4 | 27 | 31 | 5.7 | 5.6 | 8.5 | | |
| Cation | Hypotaurine | 110.0288 | 19.69 | 38830 | 35947 | 36121 | 39140 | 34976 | 34412 | 33158 | 31966 | 33165 | 34424 | 34109 | 33317 | 33485 | 31006 | 29757 | 34396 | 2.3 | 2.5 | 2.7 | 1.8 | 2.9 | 2.9 | 2.4 | 3.5 | 3.8 | 4.4 | 3.9 | 4.6 | 3.0 | 4.1 | 3.9 | 3.4 | 4.6 | 4.4 | 4.7 | 4.9 | | |
| Cation | Carnosine | 114.0627 | 8.77 | 115 | 93 | 110 | 86 | 98 | 96 | 89 | 75 | 74 | 131 | 80 | 97 | 93 | 84 | 101 | 91 | 2.8 | 2.9 | 2.6 | 2.8 | 2.9 | 2.8 | 2.3 | 2.5 | 2.6 | 2.4 | 2.6 | 2.6 | 3.3 | 3.4 | 2.7 | 3.0 | 2.9 | 2.6 | 2.9 | 2.9 | | |
| Cation | Creatinine | 114.0675 | 9.37 | 396 | 432 | 407 | 488 | 418 | 451 | 395 | 487 | 383 | 394 | 413 | 428 | 509 | 444 | 548 | 469 | 28 | 28 | 27 | 28 | 29 | 29 | 29 | 29 | 28 | 30 | 30 | 29 | 29 | 30 | 30 | 28 | 30 | 30 | 27 | 30 | | |
| Cation | Pro | 116.0726 | 13.09 | 56889 | 56697 | 55258 | 58247 | 63603 | 63488 | 60212 | 59846 | 49252 | 50089 | 50545 | 49729 | 63550 | 59270 | 60145 | 65628 | 23 | 24 | 24 | 23 | 119 | 120 | 124 | 129 | 128 | 125 | 122 | 131 | 124 | 129 | 117 | 124 | 132 | 124 | 116 | 123 | C00763 | C16435 |
| Cation | Val | 118.0887 | 12.29 | 23217 | 22500 | 22245 | 23111 | 29073 | 28937 | 27979 | 27178 | 19828 | 19208 | 19466 | 19687 | 30700 | 28036 | 28307 | 32009 | 770 | 835 | 789 | 805 | 750 | 757 | 775 | 741 | 772 | 789 | 766 | 736 | 775 | 783 | 781 | 744 | 791 | 784 | 745 | 756 | C06417 | C16436 |
| Cation | Betaine | 118.0884 | 13.55 | 1028 | 1116 | 1207 | 1295 | 1174 | 1261 | 999 | 1044 | 979 | 954 | 997 | 1045 | 1244 | 1107 | 1163 | 1212 | 23 | 26 | 23 | 22 | 28 | 29 | 28 | 30 | 31 | 32 | 31 | 28 | 24 | 25 | 25 | 25 | 24 | 25 | 25 | 26 | | |
| Cation | 5-Aminovalerate | 118.0883 | 10.20 | 319 | 309 | 351 | 301 | 356 | 356 | 273 | 244 | 305 | 330 | 277 | 291 | 324 | 277 | 307 | 386 | 0 | 0 | 0 | 0 | 0 | 0 | 0 | 0 | 0 | 0 | 0 | 0 | 0 | 0 | 0 | 0 | 0 | 0 | 0 | 0 | | |
| Cation | Thr | 120.0676 | 12.77 | 92679 | 91447 | 88890 | 93471 | 115123 | 117897 | 110344 | 109013 | 74053 | 73108 | 74237 | 74327 | 144233 | 137521 | 131943 | 146771 | 812 | 868 | 817 | 832 | 812 | 840 | 843 | 825 | 859 | 852 | 834 | 824 | 844 | 850 | 830 | 825 | 865 | 857 | 809 | 845 | C00820 | |
| Cation | Nicotinamide | 123.0576 | 9.47 | 221 | 270 | 187 | 233 | 126 | 162 | 0 | 171 | 206 | 144 | 228 | 176 | 141 | 194 | 137 | 132 | 35 | 36 | 35 | 36 | 33 | 35 | 35 | 35 | 36 | 37 | 34 | 35 | 34 | 36 | 36 | 35 | 36 | 35 | 32 | 36 | | |
| Cation | Taurine | 126.0228 | 22.56 | 3797 | 3464 | 3387 | 3054 | 2904 | 2224 | 2319 | 2601 | 3448 | 3120 | 3026 | 3319 | 2811 | 2679 | 2190 | 2733 | 17 | 16 | 15 | 17 | 12 | 11 | 12 | 12 | 14 | 14 | 15 | 12 | 12 | 4.6 | 12 | 13 | 13 | 16 | 15 | 14 | | |
| Cation | Pipecolate | 130.0873 | 12.56 | 62 | 38 | 50 | 42 | 41 | 54 | 61 | 34 | 36 | 38 | 33 | 0 | 41 | 41 | 30 | 42 | 0.27 | 0 | 0 | 0 | 0 | 0.37 | 0 | 0 | 0.32 | 0.34 | 0 | 0.37 | 0 | 0 | 0.22 | 0.30 | 0 | 0.28 | 0.19 | 0.35 | | |
| Cation | Hydroxyproline | 132.0671 | 14.29 | 1943 | 1839 | 1726 | 1969 | 2395 | 2377 | 2384 | 2318 | 1546 | 1523 | 1640 | 1553 | 3354 | 2987 | 2757 | 3359 | 8.4 | 8.9 | 9.0 | 9.3 | 18 | 18 | 18 | 18 | 18 | 11 | 18 | 19 | 19 | 19 | 20 | 19 | 19 | 19 | 18 | 19 | | |
| Cation | Creatine | 132.0788 | 11.09 | 14057 | 11492 | 12555 | 13674 | 13834 | 14135 | 13293 | 13129 | 13476 | 13737 | 14268 | 13678 | 14553 | 12940 | 13453 | 15257 | 33 | 35 | 34 | 33 | 35 | 35 | 37 | 38 | 35 | 36 | 35 | 36 | 35 | 37 | 38 | 38 | 37 | 35 | 31 | 36 | | |
| Cation | Ile | 132.1042 | 12.49 | 26237 | 25473 | 25699 | 25963 | 32269 | 33370 | 31358 | 31352 | 22331 | 22016 | 22753 | 23023 | 37776 | 33309 | 33857 | 38299 | 807 | 850 | 795 | 829 | 699 | 728 | 707 | 675 | 701 | 734 | 724 | 677 | 690 | 724 | 708 | 672 | 730 | 704 | 669 | 682 | C06418 | C16434 |
| Cation | Leu | 132.1042 | 12.59 | 26974 | 26243 | 25349 | 26579 | 34454 | 34549 | 33091 | 32825 | 22735 | 22640 | 22492 | 22577 | 38512 | 35851 | 35485 | 40177 | 745 | 801 | 764 | 780 | 663 | 671 | 674 | 670 | 692 | 693 | 679 | 662 | 705 | 705 | 705 | 672 | 705 | 714 | 664 | 678 | C01570 | C16439 |
| Cation | Asn | 133.0623 | 12.73 | 9911 | 9657 | 9721 | 9584 | 12278 | 12018 | 12092 | 11494 | 8764 | 8581 | 9067 | 8127 | 15804 | 13365 | 14694 | 16905 | 1.6 | 1.5 | 1.6 | 1.6 | 47 | 45 | 48 | 52 | 58 | 54 | 56 | 58 | 54 | 54 | 48 | 51 | 61 | 50 | 50 | 50 | C01905 | C16438 |
| Cation | Gly-Gly | 133.0622 | 10.55 | 185 | 160 | 183 | 225 | 199 | 278 | 199 | 166 | 150 | 202 | 196 | 194 | 264 | 287 | 269 | 286 | 0 | 0 | 0 | 0 | 0 | 0 | 0 | 0 | 0 | 0 | 0 | 0 | 0 | 0 | 0 | 0 | 0 | 0 | 0 | 0 | | |
| Cation | Ornithine | 133.0990 | 8.83 | 961 | 815 | 798 | 870 | 1084 | 1371 | 1505 | 1022 | 1634 | 630 | 682 | 650 | 1289 | 1347 | 1163 | 1184 | 16 | 17 | 16 | 16 | 34 | 35 | 36 | 36 | 37 | 41 | 38 | 39 | 34 | 34 | 33 | 32 | 38 | 37 | 49 | 38 | C00515 | C01602 |
| Cation | Asp | 134.0469 | 13.80 | 18118 | 17735 | 16824 | 17938 | 20101 | 20180 | 19228 | 18317 | 17902 | 18002 | 16308 | 17423 | 27679 | 27283 | 24644 | 26176 | 9.5 | 11 | 11 | 11 | 14 | 14 | 15 | 15 | 18 | 17 | 17 | 17 | 9.8 | 9.7 | 9.5 | 11 | 18 | 17 | 17 | 18 | C00402 | C16433 |
| Cation | Adenine | 136.0639 | 9.73 | 85 | 79 | 102 | 77 | 103 | 83 | 77 | 107 | 55 | 82 | 69 | 80 | 97 | 84 | 140 | 121 | 0 | 0 | 0 | 0 | 0 | 0 | 0 | 0 | 0 | 0 | 0 | 0 | 0 | 0 | 0 | 0 | 0 | 0 | 0 | 0 | | |
| Cation | Hypoxanthine | 137.0475 | 13.30 | 138 | 139 | 122 | 140 | 159 | 95 | 97 | 101 | 100 | 130 | 114 | 109 | 0 | 0 | 0 | 0 | 10 | 11 | 10 | 10 | 11 | 10 | 11 | 11 | 12 | 12 | 10 | 10 | 8.1 | 6.8 | 7.8 | 6.8 | 5.3 | 4.0 | 5.0 | 4.5 | | |
| Cation | 1-Methylnicotinamide | 137.0726 | 9.43 | 8368 | 7694 | 7926 | 8601 | 7538 | 8031 | 7433 | 6959 | 7453 | 7655 | 7510 | 7519 | 8455 | 7575 | 7954 | 8529 | 0 | 0 | 0 | 0 | 5.4 | 4.8 | 5.3 | 4.7 | 5.2 | 5.0 | 5.2 | 4.5 | 6.5 | 6.3 | 5.9 | 5.5 | 6.4 | 6.1 | 5.8 | 5.1 | | |
| Cation | gamma-Guanidinobutyrate | 146.0963 | 10.42 | 23 | 17 | 18 | 16 | 25 | 22 | 18 | 15 | 18 | 16 | 0 | 0 | 0 | 15 | 16 | 6.2 | 0 | 0 | 0 | 0 | 0 | 0 | 0 | 0 | 0 | 0 | 0 | 0 | 0 | 0 | 0 | 0 | 0 | 0 | 0 | 0 | | |
| Cation | gamma-Butyrobetaine | 146.1198 | 10.26 | 560 | 517 | 516 | 571 | 560 | 532 | 506 | 478 | 473 | 483 | 491 | 492 | 564 | 503 | 478 | 533 | 0.70 | 0.56 | 0.63 | 0.65 | 0.90 | 0.69 | 0.69 | 0.55 | 0.79 | 0.72 | 0.72 | 0.62 | 0.74 | 0.75 | 0.80 | 0.56 | 0.60 | 0.66 | 0.58 | 0.70 | | |
| Cation | Acetylcholine | 146.1189 | 9.74 | 9.5 | 13 | 9.0 | 0 | 16 | 0 | 11 | 0 | 11 | 0 | 0 | 0 | 11 | 11 | 12 | 16 | 0 | 0 | 0 | 0 | 0 | 0 | 0 | 0 | 0 | 0 | 0 | 0 | 0 | 0 | 0 | 0 | 0 | 0 | 0 | 0 | | |
| Cation | Spermidine | 146.1661 | 6.09 | 27 | 25 | 41 | 69 | 38 | 170 | 34 | 28 | 25 | 44 | 46 | 37 | 30 | 43 | 70 | 34 | 0 | 0 | 0 | 0 | 0 | 0 | 0 | 0 | 0 | 0 | 0 | 0 | 0 | 0 | 0 | 0 | 0 | 0 | 0 | 0 | | |
| Cation | Gln | 147.0792 | 12.99 | 243456 | 236964 | 235118 | 249117 | 283875 | 301252 | 277800 | 270605 | 193998 | 198872 | 196261 | 201952 | 340925 | 334796 | 325482 | 347414 | 2628 | 2745 | 2598 | 2635 | 2174 | 2187 | 2248 | 2187 | 2229 | 2227 | 2171 | 2108 | 2256 | 2300 | 2298 | 2211 | 2159 | 2186 | 2094 | 2165 | C00819 | C00303 |
| Cation | Lys | 147.1149 | 8.89 | 17606 | 16326 | 16971 | 18556 | 20402 | 23039 | 19659 | 18300 | 15745 | 15425 | 16007 | 16491 | 25837 | 24292 | 23682 | 26087 | 866 | 923 | 900 | 913 | 932 | 955 | 968 | 973 | 946 | 989 | 961 | 952 | 954 | 981 | 960 | 963 | 996 | 982 | 955 | 983 | C00739 | C16440 |
| Cation | Glu | 148.0628 | 13.20 | 262671 | 245683 | 250575 | 263784 | 242575 | 250468 | 233926 | 229289 | 254626 | 259846 | 258443 | 261922 | 299650 | 280633 | 270867 | 297930 | 85 | 97 | 91 | 91 | 249 | 240 | 249 | 262 | 284 | 291 | 283 | 297 | 220 | 219 | 217 | 224 | 348 | 315 | 304 | 318 | C00217 | C00302 |
| Cation | Met | 150.0602 | 12.97 | 8688 | 8317 | 8519 | 8668 | 11777 | 12324 | 11767 | 10998 | 7156 | 7472 | 7578 | 6889 | 15644 | 15148 | 14842 | 16322 | 187 | 197 | 190 | 188 | 187 | 183 | 194 | 191 | 192 | 198 | 195 | 189 | 190 | 201 | 198 | 188 | 200 | 200 | 186 | 195 | | |
| Cation | His | 156.0790 | 9.36 | 7933 | 8173 | 7850 | 8208 | 10722 | 10967 | 10638 | 10174 | 7851 | 7187 | 7318 | 7272 | 14094 | 12570 | 12384 | 14155 | 212 | 229 | 215 | 215 | 199 | 204 | 206 | 207 | 205 | 216 | 208 | 213 | 218 | 222 | 218 | 215 | 219 | 220 | 205 | 223 | C06419 | C00768 |
| Cation | alpha-Aminoadipate | 162.0776 | 13.23 | 3478 | 3453 | 3164 | 3408 | 3049 | 2887 | 2799 | 2885 | 3652 | 3929 | 3828 | 3648 | 2946 | 2704 | 2500 | 2930 | 2.9 | 2.9 | 2.7 | 2.8 | 4.3 | 4.1 | 3.9 | 4.6 | 4.4 | 4.3 | 4.1 | 4.8 | 4.0 | 4.1 | 4.4 | 4.5 | 4.6 | 4.2 | 4.1 | 4.7 | | |
| Cation | Carnitine | 162.1140 | 10.72 | 1261 | 1172 | 1171 | 1272 | 1245 | 1233 | 1178 | 1141 | 1214 | 1248 | 1251 | 1288 | 1392 | 1312 | 1184 | 1321 | 3.2 | 3.1 | 3.5 | 3.4 | 3.2 | 3.7 | 3.7 | 3.2 | 3.7 | 3.4 | 3.4 | 2.9 | 3.6 | 3.3 | 3.8 | 3.1 | 3.1 | 3.6 | 3.2 | 3.5 | C00487 | C15025 |
| Cation | Methionine sulfoxide | 166.0541 | 14.04 | 340 | 321 | 262 | 265 | 393 | 324 | 267 | 280 | 133 | 168 | 141 | 257 | 357 | 240 | 293 | 242 | 0 | 0 | 0 | 0 | 0 | 0 | 0 | 0 | 0 | 0 | 0 | 0 | 0 | 0 | 0 | 0 | 0 | 0 | 0 | 0 | | |
| Cation | Phe | 166.0881 | 13.37 | 19526 | 19188 | 18868 | 19427 | 25732 | 25838 | 24542 | 23705 | 17200 | 16739 | 17321 | 16512 | 30138 | 26953 | 27398 | 31569 | 444 | 449 | 441 | 429 | 433 | 439 | 452 | 435 | 458 | 447 | 446 | 433 | 453 | 458 | 449 | 446 | 459 | 455 | 422 | 442 | | |
| Cation | Pyridoxal | 168.0657 | 11.00 | 78 | 99 | 99 | 122 | 91 | 85 | 101 | 66 | 69 | 101 | 77 | 102 | 88 | 74 | 65 | 80 | 0 | 0 | 0 | 0 | 0 | 0 | 0 | 0 | 0 | 0 | 0 | 0 | 0.71 | 0 | 0 | 0.71 | 0 | 0 | 0 | 0.55 | | |
| Cation | Pyridoxine | 170.0833 | 10.85 | 296 | 290 | 278 | 309 | 293 | 325 | 309 | 294 | 251 | 283 | 244 | 285 | 372 | 340 | 299 | 340 | 31 | 30 | 30 | 30 | 26 | 26 | 26 | 23 | 25 | 27 | 25 | 24 | 29 | 31 | 31 | 27 | 29 | 31 | 28 | 27 | | |
| Cation | 3-Methylhistidine | 170.0942 | 9.58 | 270 | 278 | 249 | 320 | 365 | 374 | 344 | 340 | 261 | 261 | 236 | 268 | 510 | 410 | 394 | 461 | 7.7 | 8.3 | 7.6 | 7.9 | 6.7 | 7.5 | 7.4 | 8.0 | 7.5 | 7.1 | 6.7 | 7.9 | 7.7 | 8.6 | 8.1 | 8.4 | 9.1 | 8.4 | 7.8 | 8.7 | | |
| Cation | Arg | 175.1213 | 9.16 | 6986 | 6567 | 7088 | 7665 | 8247 | 9236 | 8096 | 7366 | 6340 | 6373 | 6597 | 6547 | 10126 | 9547 | 9519 | 10416 | 469 | 489 | 464 | 471 | 452 | 456 | 454 | 451 | 460 | 470 | 460 | 441 | 455 | 472 | 471 | 443 | 470 | 464 | 428 | 459 | C00792 | |
| Cation | Citrulline | 176.1049 | 13.32 | 240 | 254 | 234 | 234 | 373 | 369 | 310 | 289 | 236 | 262 | 248 | 254 | 409 | 371 | 429 | 400 | 8.7 | 9.7 | 9.3 | 10 | 9.0 | 8.3 | 10 | 11 | 9.3 | 10 | 10 | 9.7 | 9.2 | 9.3 | 8.0 | 9.9 | 11 | 9.5 | 9.4 | 10 | | |
| Cation | Tyr | 182.0835 | 13.62 | 19486 | 19839 | 19336 | 19595 | 25944 | 25835 | 25578 | 24502 | 17214 | 17027 | 17744 | 16725 | 31328 | 27890 | 28780 | 32199 | 410 | 421 | 411 | 406 | 408 | 417 | 415 | 409 | 433 | 421 | 414 | 409 | 431 | 421 | 431 | 421 | 435 | 431 | 399 | 417 | C06420 | C01536 |
| Cation | Phosphorylcholine | 184.0754 | 21.45 | 39120 | 35843 | 36320 | 39300 | 31414 | 31250 | 29778 | 30558 | 52860 | 52950 | 52627 | 52215 | 54323 | 48923 | 47772 | 55298 | 0 | 0 | 0 | 0 | 0 | 0 | 0 | 0 | 0 | 0 | 0 | 0 | 0 | 0 | 0 | 0 | 0 | 0 | 0 | 0 | | |
| Cation | SAM+ | 200.0788 | 9.17 | 494 | 397 | 453 | 430 | 429 | 505 | 419 | 350 | 315 | 331 | 336 | 313 | 707 | 728 | 682 | 734 | 0 | 0 | 0 | 0 | 0 | 0 | 0 | 0 | 0 | 0 | 0 | 0 | 0 | 0 | 0 | 0 | 0 | 0 | 0 | 0 | | |
| Cation | o-Acetylcarnitine | 204.1245 | 11.30 | 860 | 817 | 851 | 911 | 827 | 748 | 722 | 804 | 702 | 719 | 651 | 662 | 1053 | 952 | 940 | 1166 | 0.32 | 0.54 | 0.47 | 0.51 | 0.63 | 0.60 | 0.66 | 0.73 | 0.51 | 0.63 | 0.63 | 0.59 | 0.67 | 0.69 | 0.67 | 0.63 | 0.53 | 0.55 | 0.56 | 0.66 | | |
| Cation | Trp | 205.0992 | 13.29 | 4421 | 4518 | 4301 | 4433 | 6106 | 6218 | 6046 | 5634 | 3467 | 3430 | 3528 | 3365 | 6460 | 5905 | 6108 | 6775 | 80 | 82 | 79 | 80 | 76 | 79 | 78 | 74 | 84 | 80 | 80 | 77 | 77 | 77 | 75 | 75 | 84 | 81 | 77 | 80 | C00525 | C00806 |
| Cation | Kynurenine | 209.0939 | 12.11 | 360 | 315 | 347 | 408 | 67 | 46 | 47 | 34 | 648 | 667 | 619 | 717 | 65 | 0 | 0 | 56 | 0 | 0 | 0 | 0 | 1.9 | 2.3 | 2.2 | 2.1 | 0 | 0 | 0 | 0.81 | 4.4 | 5.4 | 4.0 | 4.3 | 0 | 0 | 0.91 | 0.72 | C01718 | |
| Cation | Cysteine-glutathione disulphide -Divalent | 214.0538 | 13.91 | 541 | 122 | 137 | 515 | 459 | 198 | 626 | 292 | 110 | 0 | 0 | 0 | 824 | 213 | 367 | 442 | 0 | 0 | 0 | 0 | 6.0 | 6.3 | 6.2 | 5.3 | 5.2 | 4.6 | 5.6 | 4.7 | 8.3 | 7.9 | 6.7 | 7.3 | 5.7 | 4.9 | 4.4 | 5.2 | | |
| Cation | Cystathionine | 223.0771 | 12.17 | 2842 | 2733 | 2718 | 2739 | 3162 | 3004 | 3033 | 3034 | 2815 | 3010 | 2865 | 2667 | 5239 | 4635 | 4672 | 5397 | 0 | 0 | 0 | 0 | 0 | 1.8 | 0 | 2.2 | 2.4 | 0 | 2.7 | 2.2 | 1.9 | 1.5 | 0 | 2.2 | 2.4 | 2.7 | 1.6 | 1.8 | C00542 | |
| Cation | Glycerophosphorylcholine | 258.1125 | 22.31 | 10015 | 10117 | 10148 | 10504 | 5642 | 4966 | 5592 | 4858 | 3300 | 3703 | 3317 | 3325 | 4544 | 4208 | 4071 | 4256 | 7.7 | 7.2 | 7.5 | 7.6 | 7.9 | 8.5 | 8.9 | 9.9 | 8.7 | 8.4 | 9.1 | 10 | 9.5 | 9.4 | 8.4 | 11 | 9.2 | 8.7 | 8.9 | 11 | | |
| Cation | Thiamine | 265.1138 | 8.61 | 924 | 795 | 885 | 957 | 878 | 1011 | 942 | 783 | 790 | 726 | 830 | 793 | 931 | 881 | 876 | 962 | 3.4 | 4.7 | 4.5 | 4.0 | 3.5 | 5.8 | 4.6 | 5.0 | 5.5 | 6.3 | 5.2 | 4.5 | 11 | 7.9 | 7.8 | 4.4 | 8.8 | 10 | 8.5 | 5.6 | | |
| Cation | Adenosine | 268.1060 | 12.13 | 393 | 457 | 382 | 439 | 293 | 720 | 307 | 331 | 259 | 168 | 251 | 190 | 254 | 237 | 352 | 477 | 0 | 0 | 0 | 0 | 0 | 0 | 0 | 0 | 0 | 0 | 0 | 0 | 0 | 0 | 0 | 0 | 0 | 0 | 0 | 0 | | |
| Cation | Inosine | 269.0893 | 20.60 | 377 | 0 | 217 | 230 | 145 | 0 | 0 | 0 | 456 | 400 | 551 | 495 | 0 | 0 | 0 | 0 | 0 | 0 | 0 | 0 | 0 | 0 | 0 | 0 | 0 | 0 | 0 | 0 | 0 | 0 | 0 | 0 | 0 | 0 | 0 | 0 | | |
| Cation | Glu-Glu | 277.1035 | 13.13 | 58 | 56 | 66 | 60 | 79 | 92 | 79 | 71 | 76 | 63 | 58 | 56 | 82 | 87 | 80 | 91 | 0 | 0 | 0 | 0 | 0 | 0 | 0 | 0 | 0 | 0 | 0 | 0 | 0 | 0 | 0 | 0 | 0 | 0 | 0 | 0 | | |
| Cation | 1-Methyladenosine | 282.1258 | 12.23 | 73 | 91 | 59 | 75 | 89 | 69 | 80 | 70 | 15 | 19 | 0 | 23 | 0 | 22 | 0 | 0 | 0 | 0 | 0 | 0 | 0 | 0 | 0 | 0 | 0 | 0 | 0 | 0 | 0 | 0 | 0 | 0 | 0 | 0 | 0 | 0 | | |
| Cation | Guanosine | 284.1014 | 14.69 | 182 | 153 | 154 | 209 | 142 | 187 | 129 | 141 | 112 | 119 | 148 | 108 | 78 | 69 | 69 | 84 | 0 | 0 | 0 | 0 | 0.66 | 0 | 0.48 | 0.72 | 0 | 0.47 | 0.51 | 0.54 | 0 | 0 | 0 | 0 | 0 | 0 | 0 | 0 | | |
| Cation | Ophthalmate | 290.1362 | 15.38 | 392 | 389 | 345 | 363 | 324 | 287 | 304 | 255 | 270 | 270 | 274 | 245 | 271 | 267 | 250 | 287 | 0 | 0 | 0 | 0 | 0 | 0 | 0 | 0 | 0 | 0 | 0 | 0 | 0 | 0 | 0 | 0 | 0 | 0 | 0 | 0 | | |
| Cation | 5-Methylthioadenosine | 298.0992 | 12.36 | 33 | 35 | 49 | 35 | 35 | 43 | 31 | 35 | 30 | 34 | 43 | 39 | 61 | 64 | 48 | 54 | 0 | 0 | 0 | 0 | 0 | 0 | 0 | 0 | 0 | 0 | 0 | 0 | 0 | 0 | 0 | 0 | 0 | 0 | 0 | 0 | | |
| Cation | Glutathione(ox) | 307.0864 | 14.42 | 22377 | 10313 | 10153 | 21019 | 17926 | 10885 | 22791 | 16834 | 8709 | 5444 | 6613 | 6856 | 27312 | 11487 | 17106 | 21431 | 0 | 0 | 0 | 0 | 0 | 0 | 0 | 0 | 0 | 0 | 0 | 0 | 0 | 0 | 0 | 0 | 0 | 0 | 0 | 0 | | |
| Cation | Glutathione(red) | 308.0940 | 15.40 | 23097 | 49737 | 47541 | 27332 | 42876 | 55510 | 25603 | 37520 | 41838 | 48560 | 46172 | 44692 | 26146 | 55203 | 43321 | 42302 | 0 | 0 | 0 | 0 | 0 | 0 | 0 | 0 | 0 | 0 | 0 | 0 | 0 | 0 | 0 | 0 | 0 | 0 | 0 | 0 | | |
| Cation | 3-Aminoisobutyrate | 104.0723 | 9.98 | 0 | 33 | 39 | 0 | 33 | 0 | 36 | 44 | 0 | 0 | 0 | 0 | 0 | 0 | 0 | 0 | 0 | 0 | 0 | 0.56 | 0.47 | 0.41 | 0 | 0.51 | 0 | 0.35 | 0 | 0 | 0.38 | 0 | 0 | 0.41 | 0.60 | 0 | 0.43 | 0.26 | | |
| Cation | N1-Acetylspermidine | 188.1758 | 8.29 | 45 | 26 | 31 | 47 | 22 | 38 | 0 | 0 | 25 | 36 | 38 | 27 | 14 | 14 | 12 | 0 | 0 | 0 | 0 | 0 | 0 | 0 | 0 | 0 | 0 | 0 | 0 | 0 | 0 | 0 | 0 | 0 | 0 | 0 | 0 | 0 | | |
| Cation | ADMA | 203.1507 | 9.80 | 29 | 0 | 39 | 36 | 50 | 41 | 40 | 29 | 0 | 54 | 29 | 33 | 36 | 46 | 40 | 61 | 0 | 0 | 0 | 0 | 0 | 0 | 0 | 0 | 0 | 0 | 0 | 0 | 0 | 0 | 0 | 0 | 0 | 0.91 | 0.77 | 0 | | |
| Cation | N1,N8-Diacetylspermidine | 230.1862 | 12.55 | 0 | 11 | 12 | 0 | 14 | 0 | 0 | 8.1 | 8.2 | 4.6 | 0 | 0 | 0 | 0 | 3.6 | 0 | 0 | 0 | 0 | 0 | 0 | 0 | 0 | 0 | 0 | 0 | 0 | 0 | 0 | 0 | 0 | 0 | 0 | 0 | 0 | 0 | | |
| Cation | Saccharopine | 277.1399 | 13.00 | 342 | 342 | 348 | 384 | 309 | 362 | 312 | 304 | 419 | 442 | 461 | 431 | 395 | 322 | 367 | 400 | 0 | 0 | 0 | 0 | 0 | 0 | 0 | 0 | 0 | 0 | 0 | 0 | 0 | 0 | 0 | 0 | 0 | 0 | 0 | 0 | | |
| Anion | Lactate | 89.0253 | 12.48 | 58173 | 61806 | 59287 | 62906 | 40830 | 41003 | 39685 | 38266 | 49278 | 43732 | 42403 | 40632 | 41540 | 40105 | 38670 | 43007 | 1546 | 1439 | 1493 | 1407 | 7592 | 7620 | 7358 | 7499 | 6464 | 6516 | 6511 | 6554 | 9023 | 8615 | 8258 | 8423 | 7859 | 7448 | 7439 | 7311 | C00256 | |
| Anion | Malonate | 103.0041 | 8.91 | 87 | 0 | 145 | 0 | 88 | 0 | 0 | 0 | 0 | 0 | 0 | 0 | 0 | 0 | 0 | 0 | 0 | 0 | 1.1 | 0 | 3.3 | 3.6 | 3.9 | 3.8 | 4.3 | 3.6 | 3.9 | 3.4 | 1.5 | 1.7 | 0.84 | 0 | 1.0 | 1.1 | 0 | 0 | | |
| Anion | 3-Hydroxybutyrate | 103.0407 | 13.69 | 331 | 313 | 371 | 294 | 396 | 323 | 323 | 361 | 215 | 233 | 300 | 253 | 276 | 327 | 238 | 295 | 18 | 18 | 18 | 19 | 24 | 25 | 25 | 25 | 25 | 27 | 25 | 23 | 21 | 24 | 22 | 22 | 23 | 23 | 26 | 21 | C03197 | |
| Anion | 2-Hydroxybutyrate | 103.0405 | 13.47 | 138 | 145 | 105 | 135 | 138 | 0 | 81 | 0 | 0 | 0 | 0 | 0 | 0 | 0 | 0 | 0 | 6.1 | 5.3 | 6.4 | 5.1 | 32 | 31 | 35 | 33 | 35 | 34 | 31 | 32 | 7.2 | 6.7 | 6.9 | 6.4 | 7.2 | 7.5 | 8.0 | 7.0 | | |
| Anion | Fumarate | 115.0044 | 9.25 | 1527 | 1447 | 1630 | 1484 | 1106 | 1111 | 927 | 944 | 1443 | 1574 | 1484 | 1440 | 1555 | 1427 | 1514 | 1136 | 6.6 | 5.9 | 6.4 | 5.5 | 6.9 | 6.6 | 7.3 | 6.7 | 7.0 | 5.0 | 5.9 | 5.5 | 5.0 | 7.0 | 6.7 | 6.0 | 5.1 | 5.5 | 4.5 | 5.6 | | |
| Anion | 2-Oxoisopentanoate | 115.0405 | 13.07 | 284 | 215 | 281 | 191 | 202 | 313 | 195 | 186 | 109 | 206 | 235 | 205 | 224 | 235 | 176 | 238 | 0 | 0 | 0 | 0 | 65 | 68 | 68 | 71 | 77 | 74 | 69 | 76 | 71 | 72 | 67 | 66 | 75 | 66 | 63 | 60 | | |
| Anion | Hexanoate | 115.0767 | 14.56 | 183 | 107 | 150 | 140 | 135 | 162 | 170 | 141 | 111 | 111 | 132 | 109 | 122 | 138 | 137 | 134 | 0 | 2.1 | 2.3 | 2.5 | 3.5 | 3.4 | 3.5 | 2.7 | 2.9 | 2.9 | 2.5 | 2.2 | 3.3 | 2.6 | 3.2 | 2.7 | 2.6 | 3.7 | 0 | 2.6 | | |
| Anion | Succinate | 117.0200 | 9.61 | 1110 | 1149 | 1041 | 1134 | 725 | 811 | 701 | 675 | 983 | 809 | 762 | 746 | 753 | 692 | 792 | 882 | 92 | 87 | 89 | 82 | 89 | 93 | 89 | 89 | 90 | 91 | 85 | 88 | 88 | 88 | 90 | 85 | 90 | 89 | 86 | 88 | | |
| Anion | 2-Hydroxypentanoate | 117.0559 | 14.25 | 341 | 235 | 298 | 303 | 261 | 294 | 270 | 163 | 222 | 222 | 221 | 208 | 188 | 235 | 193 | 295 | 5.4 | 1.3 | 5.4 | 5.9 | 9.3 | 9.5 | 8.2 | 14 | 8.7 | 13 | 5.7 | 14 | 7.5 | 5.2 | 13 | 10 | 9.6 | 9.9 | 12 | 8.7 | | |
| Anion | 5-Oxoproline | 128.0358 | 13.73 | 9583 | 9200 | 9523 | 9723 | 8653 | 8849 | 8230 | 7394 | 9073 | 7334 | 7671 | 7127 | 9142 | 8571 | 8370 | 9140 | 1360 | 1329 | 1425 | 1315 | 1489 | 1554 | 1549 | 1500 | 1551 | 1550 | 1623 | 1539 | 1564 | 1546 | 1527 | 1527 | 1561 | 1584 | 1567 | 1537 | | |
| Anion | Citraconate | 129.0200 | 10.16 | 22 | 20 | 0 | 0 | 0 | 0 | 0 | 0 | 0 | 0 | 0 | 0 | 0 | 15 | 0 | 0 | 0.31 | 0 | 0.43 | 0.36 | 0 | 0 | 0.29 | 0 | 0 | 0 | 0 | 0 | 0 | 0 | 0.25 | 0.27 | 0.46 | 0.38 | 0 | 0.35 | | |
| Anion | 4-Methyl-2-oxopentanoate | 129.0556 | 13.69 | 1163 | 1216 | 1179 | 1173 | 987 | 1016 | 978 | 846 | 805 | 834 | 1052 | 785 | 997 | 845 | 753 | 950 | 0 | 0 | 0 | 0 | 317 | 315 | 313 | 326 | 335 | 329 | 321 | 342 | 329 | 329 | 314 | 323 | 358 | 335 | 336 | 326 | | |
| Anion | N-Acetyl-beta-alanine | 130.0515 | 14.54 | 554 | 368 | 370 | 511 | 403 | 360 | 359 | 381 | 299 | 286 | 328 | 265 | 300 | 339 | 278 | 322 | 0 | 0 | 0 | 0 | 0.96 | 0 | 0 | 0 | 0 | 0 | 0 | 0 | 0 | 0 | 0 | 0 | 0 | 0 | 0 | 0 | | |
| Anion | Glutarate | 131.0361 | 10.20 | 152 | 161 | 165 | 174 | 0 | 0 | 0 | 0 | 135 | 151 | 0 | 77 | 111 | 0 | 158 | 96 | 4.3 | 3.3 | 4.6 | 5.6 | 3.7 | 4.6 | 3.6 | 3.9 | 4.9 | 4.9 | 5.5 | 4.3 | 4.0 | 3.2 | 4.7 | 4.4 | 4.4 | 3.6 | 4.8 | 4.3 | | |
| Anion | Malate | 133.0148 | 9.57 | 5671 | 5726 | 5763 | 6031 | 3711 | 3808 | 3406 | 3242 | 6237 | 6080 | 5590 | 5518 | 5145 | 5255 | 5079 | 5166 | 13 | 13 | 14 | 14 | 23 | 24 | 23 | 23 | 26 | 23 | 23 | 22 | 15 | 18 | 16 | 17 | 15 | 16 | 15 | 16 | C00149 | C00497 |
| Anion | Ethanolamine phosphate | 140.0117 | 16.30 | 0 | 131 | 102 | 0 | 116 | 0 | 0 | 0 | 0 | 143 | 0 | 151 | 0 | 0 | 0 | 157 | 0 | 0 | 0 | 0 | 0 | 0 | 0 | 0 | 0 | 0 | 0 | 0 | 0 | 0 | 0 | 0 | 0 | 0 | 0 | 0 | | |
| Anion | 2-Oxoglutarate | 145.0141 | 9.54 | 1159 | 1200 | 1147 | 1075 | 656 | 771 | 862 | 736 | 1182 | 1066 | 1179 | 1110 | 549 | 559 | 625 | 659 | 0 | 0 | 0 | 0 | 9.8 | 11 | 11 | 10 | 11 | 10 | 10 | 11 | 12 | 11 | 12 | 9.7 | 11 | 12 | 13 | 8.7 | | |
| Anion | Adipate | 145.0506 | 10.80 | 86 | 79 | 158 | 177 | 108 | 100 | 78 | 52 | 57 | 73 | 44 | 86 | 70 | 90 | 75 | 104 | 1.0 | 1.2 | 1.1 | 0.81 | 0.69 | 1.2 | 0.74 | 1.0 | 0.86 | 0.72 | 0.86 | 0.98 | 0.66 | 0.53 | 0.80 | 0.95 | 0.49 | 0.84 | 0.83 | 1.0 | | |
| Anion | 2-Hydroxyglutarate | 147.0305 | 10.19 | 420 | 398 | 376 | 405 | 217 | 211 | 239 | 269 | 406 | 481 | 358 | 375 | 324 | 282 | 223 | 351 | 2.7 | 3.0 | 2.3 | 3.0 | 6.6 | 5.5 | 8.8 | 5.2 | 6.5 | 10 | 5.8 | 5.0 | 2.6 | 3.7 | 2.0 | 2.2 | 2.6 | 1.9 | 2.1 | 2.1 | C01087 | C03196 |
| Anion | trans-Cinnamate | 147.0499 | 14.71 | 7129 | 7192 | 6822 | 7069 | 6866 | 6644 | 6675 | 6443 | 7205 | 7551 | 7360 | 7335 | 8502 | 8212 | 7805 | 8463 | 3.1 | 3.6 | 3.4 | 4.4 | 9.3 | 9.7 | 9.8 | 10 | 9.6 | 10 | 10 | 9.1 | 9.1 | 7.1 | 9.3 | 7.5 | 13 | 11 | 11 | 9.1 | | |
| Anion | Cysteine sulfinate | 152.0028 | 13.66 | 849 | 912 | 951 | 823 | 1386 | 1520 | 1276 | 1149 | 834 | 804 | 933 | 745 | 2128 | 2167 | 1867 | 2263 | 2.6 | 2.6 | 0 | 0 | 0 | 0 | 0 | 0 | 0 | 0 | 2.3 | 3.1 | 0 | 0 | 0 | 0 | 0 | 2.6 | 0 | 0 | | |
| Anion | Pelargonate | 157.1235 | 16.10 | 253 | 186 | 185 | 0 | 166 | 315 | 285 | 131 | 191 | 250 | 123 | 198 | 158 | 214 | 200 | 180 | 3.7 | 0 | 0 | 2.6 | 0 | 0 | 0 | 2.7 | 0 | 0 | 0 | 2.2 | 3.0 | 0 | 0 | 2.5 | 0 | 2.8 | 3.3 | 3.0 | | |
| Anion | 3-Hydroxy-3-methylglutarate | 161.0458 | 10.62 | 304 | 271 | 334 | 285 | 232 | 244 | 240 | 248 | 112 | 139 | 150 | 136 | 100 | 138 | 131 | 128 | 0 | 0 | 0 | 0 | 0 | 0 | 0 | 1.7 | 0 | 0 | 0 | 0 | 0 | 0 | 0 | 0 | 0 | 0 | 0 | 0 | | |
| Anion | p-Coumarate | 163.0403 | 14.71 | 532 | 452 | 469 | 593 | 358 | 432 | 289 | 250 | 0 | 0 | 0 | 0 | 0 | 0 | 0 | 0 | 0 | 0 | 0 | 0 | 128 | 143 | 142 | 141 | 163 | 153 | 136 | 131 | 0 | 0 | 0 | 0 | 0 | 0 | 0 | 0 | | |
| Anion | PEP | 166.9768 | 9.61 | 86 | 89 | 94 | 82 | 74 | 0 | 100 | 0 | 69 | 75 | 0 | 0 | 0 | 72 | 93 | 71 | 0 | 0 | 0 | 0 | 0 | 0 | 0 | 0 | 0 | 0 | 0 | 0 | 0 | 0 | 0 | 0 | 0 | 0 | 0 | 0 | | |
| Anion | Cysteate | 167.9980 | 12.21 | 193 | 145 | 105 | 156 | 152 | 168 | 145 | 166 | 180 | 188 | 161 | 181 | 160 | 137 | 121 | 123 | 0 | 0 | 0 | 0 | 0 | 0 | 0 | 0 | 0 | 0 | 0 | 0 | 0 | 0 | 0 | 0 | 0 | 0 | 0 | 0 | | |
| Anion | DHAP | 168.9913 | 11.51 | 5549 | 5008 | 5451 | 5912 | 4037 | 4108 | 4924 | 4273 | 7673 | 5896 | 6522 | 5999 | 5074 | 5444 | 5277 | 5937 | 0 | 0 | 0 | 0 | 0 | 0 | 0 | 0 | 0 | 0 | 0 | 0 | 0 | 0 | 0 | 0 | 0 | 0 | 0 | 0 | | |
| Anion | Glycerophosphate | 171.0076 | 11.72 | 1010 | 835 | 914 | 966 | 915 | 896 | 967 | 716 | 998 | 762 | 1058 | 895 | 1166 | 979 | 837 | 949 | 2.1 | 2.8 | 0 | 2.6 | 3.9 | 3.1 | 3.2 | 2.6 | 2.7 | 3.9 | 3.2 | 3.6 | 2.8 | 0 | 0 | 1.6 | 1.6 | 0 | 2.5 | 0 | C03189 | |
| Anion | cis-Aconitate | 173.0100 | 9.09 | 548 | 542 | 543 | 554 | 310 | 356 | 292 | 305 | 578 | 509 | 499 | 523 | 610 | 586 | 639 | 675 | 0.73 | 0.91 | 0.67 | 0.67 | 1.2 | 1.4 | 1.2 | 1.2 | 1.3 | 1.1 | 1.2 | 1.1 | 1.1 | 1.1 | 1.0 | 1.0 | 0.98 | 1.2 | 1.0 | 0.85 | | |
| Anion | N-Acetylaspartate | 174.0411 | 10.84 | 1597 | 1480 | 1424 | 1498 | 1527 | 1370 | 1523 | 1543 | 1323 | 1160 | 1265 | 1155 | 1562 | 1349 | 1446 | 1544 | 0.33 | 0.31 | 0.45 | 0 | 0.62 | 0.42 | 0.73 | 0.45 | 0.37 | 0.32 | 0.41 | 0.61 | 0.62 | 0.57 | 0.39 | 0.58 | 0.82 | 0.82 | 0.55 | 0.70 | | |
| Anion | O-Phosphoserine | 184.0020 | 11.66 | 683 | 607 | 633 | 645 | 621 | 690 | 767 | 640 | 352 | 427 | 324 | 369 | 390 | 541 | 423 | 435 | 0 | 0 | 0 | 0 | 0 | 0 | 0 | 0 | 0 | 0 | 0 | 0 | 0 | 0 | 0 | 0 | 0 | 0 | 0 | 0 | | |
| Anion | 3PG | 184.9865 | 9.77 | 751 | 502 | 547 | 754 | 365 | 478 | 419 | 286 | 481 | 469 | 473 | 527 | 485 | 404 | 446 | 483 | 0 | 0 | 0 | 0 | 0 | 0 | 0 | 0 | 0 | 0 | 0 | 0 | 0 | 0 | 0 | 0 | 0 | 0 | 0 | 0 | C00597 | |
| Anion | Azelate | 187.0985 | 11.75 | 50 | 0 | 34 | 39 | 50 | 85 | 47 | 0 | 30 | 43 | 29 | 48 | 26 | 0 | 50 | 43 | 0 | 0 | 0 | 0.28 | 0.55 | 0.64 | 0.88 | 0.55 | 0.66 | 0.93 | 0.79 | 0.61 | 0 | 0.57 | 0.37 | 0.36 | 0.46 | 0 | 0.39 | 0.57 | | |
| Anion | N-Acetylglutamate | 188.0558 | 11.26 | 284 | 261 | 372 | 323 | 259 | 199 | 251 | 210 | 185 | 184 | 218 | 200 | 237 | 215 | 257 | 289 | 0 | 0 | 0 | 0.90 | 0 | 0 | 0 | 0 | 0 | 0 | 0 | 1.6 | 0 | 0 | 1.1 | 1.2 | 0 | 0 | 0 | 0.89 | | |
| Anion | Isocitrate | 191.0244 | 9.06 | 798 | 510 | 424 | 617 | 469 | 275 | 475 | 347 | 527 | 356 | 426 | 482 | 633 | 488 | 420 | 504 | 0 | 0 | 0 | 0 | 3.1 | 3.5 | 2.0 | 0 | 4.4 | 2.4 | 1.2 | 3.2 | 0 | 2.9 | 0 | 1.9 | 1.4 | 2.2 | 0 | 0 | | |
| Anion | Citrate | 191.0204 | 9.24 | 9726 | 9810 | 9270 | 9992 | 6213 | 6590 | 6002 | 5670 | 9367 | 9360 | 9688 | 9377 | 11548 | 11160 | 11204 | 12327 | 18 | 18 | 18 | 18 | 31 | 32 | 31 | 32 | 33 | 32 | 31 | 32 | 25 | 25 | 25 | 25 | 28 | 26 | 25 | 27 | | |
| Anion | Syringate | 197.0488 | 15.18 | 166 | 198 | 240 | 154 | 297 | 247 | 0 | 186 | 243 | 260 | 188 | 257 | 222 | 0 | 193 | 309 | 0 | 0 | 0 | 0 | 0 | 0 | 0 | 0 | 0 | 0 | 0 | 0 | 0 | 0 | 0 | 0 | 0 | 0 | 0 | 0 | | |
| Anion | Dodecanoate | 199.1711 | 17.80 | 671 | 575 | 639 | 702 | 695 | 635 | 573 | 654 | 620 | 585 | 650 | 656 | 551 | 646 | 596 | 602 | 6.2 | 6.0 | 5.7 | 5.7 | 5.1 | 5.3 | 5.6 | 5.3 | 5.8 | 5.7 | 4.3 | 5.2 | 5.6 | 4.6 | 5.1 | 5.2 | 4.9 | 6.0 | 8.5 | 4.7 | | |
| Anion | Pantothenate | 218.1039 | 17.16 | 3850 | 3828 | 3806 | 4074 | 4376 | 3966 | 4125 | 3686 | 2706 | 2645 | 2644 | 2598 | 3452 | 3144 | 3074 | 3130 | 23 | 24 | 22 | 23 | 22 | 24 | 23 | 24 | 24 | 24 | 24 | 23 | 24 | 24 | 24 | 23 | 25 | 23 | 24 | 23 | | |
| Anion | R5P | 229.0121 | 12.74 | 293 | 212 | 295 | 332 | 205 | 213 | 231 | 188 | 242 | 276 | 241 | 272 | 204 | 217 | 216 | 266 | 0 | 0 | 0 | 0 | 0 | 0 | 0 | 0 | 0 | 0 | 0 | 0 | 0 | 0 | 0 | 0 | 0 | 0 | 0 | 0 | C03736 | |
| Anion | Ru5P | 229.0121 | 12.48 | 1217 | 1047 | 790 | 1008 | 707 | 788 | 691 | 609 | 1195 | 1002 | 1081 | 911 | 1089 | 886 | 855 | 1014 | 0 | 0 | 0 | 0 | 0 | 0 | 0 | 0 | 0 | 0 | 0 | 0 | 0 | 0 | 0 | 0 | 0 | 0 | 0 | 0 | C01101 | |
| Anion | Biotin | 243.0836 | 17.20 | 1838 | 1734 | 1813 | 1815 | 1424 | 1592 | 1533 | 1443 | 2356 | 2510 | 2590 | 2342 | 2397 | 2270 | 2315 | 2631 | 0 | 0 | 0 | 0 | 0 | 0 | 0 | 0 | 0 | 0 | 0 | 0 | 0 | 0 | 0 | 0 | 0 | 0 | 0 | 0 | | |
| Anion | G6P | 259.0230 | 13.42 | 2582 | 2613 | 2911 | 3134 | 2597 | 2833 | 2754 | 2450 | 2889 | 2682 | 2468 | 2676 | 4331 | 4186 | 4080 | 4386 | 0 | 0 | 0 | 0 | 0 | 0 | 0 | 0 | 0 | 0 | 0 | 0 | 0 | 0 | 0 | 0 | 0 | 0 | 0 | 0 | C00668 | |
| Anion | F6P | 259.0227 | 13.30 | 896 | 543 | 706 | 879 | 814 | 585 | 674 | 665 | 778 | 745 | 763 | 1024 | 1243 | 897 | 1095 | 1068 | 0 | 0 | 0 | 0 | 0 | 0 | 0 | 0 | 0 | 0 | 0 | 0 | 0 | 0 | 0 | 0 | 0 | 0 | 0 | 0 | C05345 | |
| Anion | G1P | 259.0227 | 13.16 | 701 | 624 | 522 | 621 | 362 | 332 | 285 | 285 | 518 | 650 | 649 | 703 | 512 | 468 | 422 | 482 | 0 | 0 | 0 | 0 | 0 | 0 | 0 | 0 | 0 | 0 | 0 | 0 | 0 | 0 | 0 | 0 | 0 | 0 | 0 | 0 | C00663 | |
| Anion | 2,3-DPG | 264.9522 | 9.98 | 1277 | 1088 | 888 | 1369 | 446 | 508 | 486 | 351 | 1228 | 1055 | 1282 | 1364 | 791 | 655 | 788 | 702 | 0 | 0 | 0 | 0 | 0 | 0 | 0 | 0 | 0 | 0 | 0 | 0 | 0 | 0 | 0 | 0 | 0 | 0 | 0 | 0 | C03339 | |
| Anion | 6-Phosphogluconate | 275.0176 | 10.79 | 1006 | 981 | 901 | 1049 | 719 | 707 | 711 | 613 | 893 | 899 | 868 | 955 | 1214 | 1137 | 1103 | 1170 | 0 | 0 | 0 | 0 | 0 | 0 | 0 | 0 | 0 | 0 | 0 | 0 | 0 | 0 | 0 | 0 | 0 | 0 | 0 | 0 | | |
| Anion | S7P | 289.0341 | 13.63 | 449 | 490 | 385 | 438 | 346 | 369 | 340 | 332 | 451 | 467 | 499 | 437 | 395 | 375 | 443 | 408 | 0 | 0 | 0 | 0 | 0 | 0 | 0 | 0 | 0 | 0 | 0 | 0 | 0 | 0 | 0 | 0 | 0 | 0 | 0 | 0 | | |
| Anion | Argininosuccinate | 289.1160 | 18.24 | 201 | 221 | 247 | 217 | 137 | 0 | 145 | 154 | 244 | 285 | 191 | 197 | 263 | 222 | 264 | 278 | 0 | 0 | 0 | 0 | 0 | 0 | 0 | 0 | 0 | 0 | 0 | 0 | 0 | 0 | 0 | 0 | 0 | 0 | 0 | 0 | | |
| Anion | N-Acetylglucosamine 6-phosphate | 300.0489 | 14.21 | 93 | 81 | 91 | 77 | 163 | 209 | 169 | 129 | 73 | 95 | 86 | 76 | 185 | 146 | 178 | 127 | 0 | 0 | 0 | 0 | 0 | 0 | 0 | 0 | 0 | 0 | 0 | 0 | 0 | 0 | 0 | 0 | 0 | 0 | 0 | 0 | | |
| Anion | N-Acetylglucosamine 1-phosphate | 300.0491 | 13.73 | 299 | 227 | 248 | 309 | 313 | 386 | 297 | 187 | 254 | 338 | 255 | 292 | 280 | 343 | 353 | 335 | 0 | 0 | 0 | 0 | 0 | 0 | 0 | 0 | 0 | 0 | 0 | 0 | 0 | 0 | 0 | 0 | 0 | 0 | 0 | 0 | C04256 | |
| Anion | N-Acetylneuraminate | 308.1031 | 18.75 | 348 | 389 | 338 | 400 | 314 | 267 | 261 | 235 | 448 | 390 | 435 | 413 | 571 | 477 | 434 | 557 | 56 | 55 | 49 | 57 | 43 | 46 | 41 | 43 | 45 | 43 | 41 | 59 | 49 | 45 | 45 | 62 | 45 | 38 | 40 | 53 | | |
| Anion | Ribulose 1,5-diphosphate | 308.9781 | 10.51 | 80 | 0 | 89 | 101 | 0 | 0 | 0 | 58 | 81 | 75 | 85 | 87 | 0 | 0 | 0 | 0 | 0 | 0 | 0 | 0 | 0 | 0 | 0 | 0 | 0 | 0 | 0 | 0 | 0 | 0 | 0 | 0 | 0 | 0 | 0 | 0 | C01151 | |
| Anion | UMP | 323.0298 | 13.46 | 284 | 186 | 231 | 324 | 157 | 275 | 136 | 138 | 295 | 248 | 365 | 327 | 233 | 180 | 211 | 191 | 0 | 0 | 0 | 0 | 0 | 0 | 0 | 0 | 0 | 0 | 0 | 0 | 0 | 0 | 0 | 0 | 0 | 0 | 0 | 0 | | |
| Anion | F1,6P | 338.9889 | 10.81 | 10066 | 7843 | 7555 | 9224 | 4724 | 4794 | 4613 | 4112 | 12456 | 12412 | 14246 | 13188 | 5851 | 5631 | 6042 | 6130 | 0 | 0 | 0 | 0 | 0 | 0 | 0 | 0 | 0 | 0 | 0 | 0 | 0 | 0 | 0 | 0 | 0 | 0 | 0 | 0 | C05378 | |
| Anion | AMP | 346.0563 | 14.04 | 521 | 360 | 400 | 412 | 400 | 245 | 340 | 346 | 600 | 526 | 673 | 523 | 370 | 415 | 475 | 441 | 0 | 0 | 0 | 0 | 0 | 0 | 0 | 0 | 0 | 0 | 0 | 0 | 0 | 0 | 0 | 0 | 0 | 0 | 0 | 0 | | |
| Anion | GMP | 362.0510 | 14.19 | 230 | 147 | 184 | 100 | 197 | 0 | 161 | 134 | 196 | 168 | 220 | 208 | 196 | 141 | 146 | 164 | 0 | 0 | 0 | 0 | 0 | 0 | 0 | 0 | 0 | 0 | 0 | 0 | 0 | 0 | 0 | 0 | 0 | 0 | 0 | 0 | | |
| Anion | NADPH | 371.5384 | 12.23 | 305 | 220 | 180 | 212 | 187 | 208 | 157 | 172 | 343 | 344 | 356 | 314 | 296 | 271 | 307 | 365 | 0 | 0 | 0 | 0 | 0 | 0 | 0 | 0 | 0 | 0 | 0 | 0 | 0 | 0 | 0 | 0 | 0 | 0 | 0 | 0 | | |
| Anion | CoA | 382.5481 | 12.73 | 48 | 0 | 61 | 0 | 47 | 119 | 0 | 63 | 93 | 68 | 134 | 87 | 99 | 72 | 110 | 113 | 0 | 0 | 0 | 0 | 0 | 0 | 0 | 0 | 0 | 0 | 0 | 0 | 0 | 0 | 0 | 0 | 0 | 0 | 0 | 0 | | |
| Anion | CDP | 402.0108 | 12.11 | 349 | 199 | 259 | 250 | 353 | 372 | 251 | 284 | 332 | 294 | 444 | 312 | 180 | 185 | 222 | 227 | 0 | 0 | 0 | 0 | 0 | 0 | 0 | 0 | 0 | 0 | 0 | 0 | 0 | 0 | 0 | 0 | 0 | 0 | 0 | 0 | | |
| Anion | UDP | 402.9950 | 11.97 | 699 | 506 | 499 | 667 | 515 | 580 | 447 | 481 | 674 | 703 | 962 | 814 | 454 | 431 | 565 | 662 | 0 | 0 | 0 | 0 | 0 | 0 | 0 | 0 | 0 | 0 | 0 | 0 | 0 | 0 | 0 | 0 | 0 | 0 | 0 | 0 | | |
| Anion | ADP | 426.0224 | 12.53 | 4174 | 3521 | 3228 | 3995 | 3642 | 3532 | 3362 | 3287 | 4392 | 4369 | 5261 | 4913 | 3970 | 3659 | 4308 | 4360 | 0 | 0 | 0 | 0 | 0 | 0 | 0 | 0 | 0 | 0 | 0 | 0 | 0 | 0 | 0 | 0 | 0 | 0 | 0 | 0 | | |
| Anion | GDP | 442.0176 | 12.70 | 885 | 673 | 704 | 843 | 767 | 844 | 635 | 607 | 852 | 849 | 880 | 863 | 716 | 724 | 785 | 763 | 0 | 0 | 0 | 0 | 0 | 0 | 0 | 0 | 0 | 0 | 0 | 0 | 0 | 0 | 0 | 0 | 0 | 0 | 0 | 0 | | |
| Anion | CTP | 481.9774 | 11.71 | 4913 | 4590 | 4591 | 5698 | 5050 | 5798 | 5175 | 4581 | 4731 | 4397 | 4428 | 4783 | 4681 | 4235 | 4742 | 4789 | 0 | 0 | 0 | 0 | 0 | 0 | 0 | 0 | 0 | 0 | 0 | 0 | 0 | 0 | 0 | 0 | 0 | 0 | 0 | 0 | | |
| Anion | UTP | 482.9615 | 11.60 | 10282 | 9717 | 8962 | 11358 | 7626 | 8281 | 7876 | 6908 | 11062 | 10331 | 9484 | 9906 | 10608 | 10672 | 11014 | 11328 | 0 | 0 | 0 | 0 | 0 | 0 | 0 | 0 | 0 | 0 | 0 | 0 | 0 | 0 | 0 | 0 | 0 | 0 | 0 | 0 | | |
| Anion | ATP | 505.9886 | 11.97 | 46485 | 44554 | 43426 | 52683 | 45892 | 52836 | 46671 | 42223 | 47861 | 45721 | 46843 | 46686 | 63317 | 63239 | 65954 | 65813 | 0 | 0 | 0 | 0 | 0 | 0 | 0 | 0 | 0 | 0 | 0 | 0 | 0 | 0 | 0 | 0 | 0 | 0 | 0 | 0 | | |
| Anion | ITP | 506.9889 | 11.96 | 4561 | 4372 | 4137 | 4979 | 4478 | 4845 | 4266 | 4170 | 4621 | 4223 | 4650 | 4550 | 6103 | 5937 | 6294 | 6234 | 0 | 0 | 0 | 0 | 0 | 0 | 0 | 0 | 0 | 0 | 0 | 0 | 0 | 0 | 0 | 0 | 0 | 0 | 0 | 0 | | |
| Anion | GTP | 521.9833 | 12.23 | 13216 | 12888 | 13562 | 16760 | 12534 | 18308 | 13385 | 11294 | 11960 | 13301 | 12980 | 12681 | 14312 | 14864 | 16598 | 14062 | 0 | 0 | 0 | 0 | 0 | 0 | 0 | 0 | 0 | 0 | 0 | 0 | 0 | 0 | 0 | 0 | 0 | 0 | 0 | 0 | | |
| Anion | UDP-glucose | 565.0481 | 14.98 | 6867 | 6217 | 6085 | 6888 | 4017 | 4393 | 4366 | 4014 | 7154 | 7061 | 7219 | 7072 | 6221 | 5677 | 6197 | 6663 | 0 | 0 | 0 | 0 | 0 | 0 | 0 | 0 | 0 | 0 | 0 | 0 | 0 | 0 | 0 | 0 | 0 | 0 | 0 | 0 | | |
| Anion | UDP-glucuronate | 579.0274 | 12.42 | 782 | 741 | 640 | 872 | 716 | 968 | 540 | 532 | 824 | 897 | 889 | 1055 | 1061 | 1052 | 1229 | 992 | 0 | 0 | 0 | 0 | 0 | 0 | 0 | 0 | 0 | 0 | 0 | 0 | 0 | 0 | 0 | 0 | 0 | 0 | 0 | 0 | | |
| Anion | ADP-glucose | 588.0745 | 15.60 | 376 | 349 | 341 | 434 | 359 | 433 | 379 | 351 | 343 | 359 | 396 | 353 | 369 | 407 | 343 | 475 | 0 | 0 | 0 | 0 | 0 | 0 | 0 | 0 | 0 | 0 | 0 | 0 | 0 | 0 | 0 | 0 | 0 | 0 | 0 | 0 | | |
| Anion | GDP-mannose | 604.0690 | 15.65 | 218 | 174 | 136 | 188 | 168 | 258 | 204 | 181 | 176 | 178 | 153 | 201 | 208 | 214 | 226 | 226 | 0 | 0 | 0 | 0 | 0 | 0 | 0 | 0 | 0 | 0 | 0 | 0 | 0 | 0 | 0 | 0 | 0 | 0 | 0 | 0 | | |
| Anion | UDP-N-acetylglucosamine | 606.0744 | 15.27 | 20636 | 17906 | 15728 | 19284 | 26963 | 29288 | 26696 | 19643 | 17430 | 19033 | 17892 | 20834 | 30556 | 30759 | 29581 | 23533 | 0 | 0 | 0 | 0 | 0 | 0 | 0 | 0 | 0 | 0 | 0 | 0 | 0 | 0 | 0 | 0 | 0 | 0 | 0 | 0 | | |
| Anion | CMP-N-acetylneuraminate | 613.1395 | 15.94 | 403 | 385 | 436 | 396 | 310 | 323 | 311 | 294 | 494 | 473 | 539 | 553 | 547 | 458 | 537 | 527 | 0 | 0 | 0 | 0 | 0 | 0 | 0 | 0 | 0 | 0 | 0 | 0 | 0 | 0 | 0 | 0 | 0 | 0 | 0 | 0 | | |
| Anion | NAD+ | 662.1016 | 21.75 | 4961 | 4831 | 5102 | 5361 | 4534 | 4838 | 4651 | 4480 | 5426 | 5522 | 5109 | 5056 | 5759 | 5620 | 5773 | 6124 | 0 | 0 | 0 | 0 | 0 | 0 | 0 | 0 | 0 | 0 | 0 | 0 | 0 | 0 | 0 | 0 | 0 | 0 | 0 | 0 | | |
| Anion | NADH | 664.1181 | 15.88 | 630 | 551 | 562 | 665 | 399 | 505 | 409 | 423 | 595 | 667 | 766 | 563 | 692 | 560 | 592 | 657 | 0 | 0 | 0 | 0 | 0 | 0 | 0 | 0 | 0 | 0 | 0 | 0 | 0 | 0 | 0 | 0 | 0 | 0 | 0 | 0 | | |
| Anion | Threonate | 135.0325 | 14.13 | 1270 | 1765 | 1258 | 1534 | 1125 | 1072 | 1208 | 1096 | 1040 | 1360 | 1096 | 1176 | 1123 | 1205 | 1419 | 1191 | 33 | 0 | 17 | 37 | 30 | 29 | 0 | 0 | 0 | 26 | 35 | 33 | 0 | 0 | 32 | 0 | 0 | 0 | 0 | 0 | | |
| Anion | G3P | 168.9916 | 12.02 | 1173 | 745 | 727 | 1007 | 505 | 910 | 359 | 417 | 304 | 923 | 970 | 661 | 668 | 528 | 850 | 810 | 0 | 0 | 0 | 0 | 0 | 0 | 0 | 0 | 0 | 0 | 0 | 0 | 0 | 0 | 0 | 0 | 0 | 0 | 0 | 0 | C00118 | |
| Anion | 2PG | 184.9866 | 9.82 | 132 | 124 | 148 | 134 | 0 | 0 | 0 | 0 | 78 | 83 | 83 | 0 | 97 | 0 | 0 | 0 | 0 | 0 | 0 | 0 | 0 | 0 | 0 | 0 | 0 | 0 | 0 | 0 | 0 | 0 | 0 | 0 | 0 | 0 | 0 | 0 | | |
| Anion | threo-beta-methylaspartate+ Glu | 146.0467 | 14.71 | 203598 | 196463 | 190402 | 198646 | 194755 | 182234 | 189263 | 180618 | 219263 | 211065 | 211565 | 205659 | 246681 | 232655 | 224303 | 243560 | 82 | 77 | 79 | 80 | 212 | 213 | 212 | 219 | 237 | 231 | 231 | 200 | 189 | 178 | 181 | 149 | 268 | 240 | 264 | 203 | C00217 | C00302 |

# Supplementary Table S2

## Original data with SD values for Table 1

| | SE+IL-1β | | | | SE | | | | IL-1β | | | | Control | | |
|---|---|---|---|---|---|---|---|---|---|---|---|---|---|---|---|
| | mean | SD | % of control | | mean | SD | % of control | | | | | | mean | SD | % of contro |
| p-Coumara | 511.4104 | 64.15936 | | | 332.4037 | 80.18073 | | | 0 | 0 | | | 0 | 0 | |
| Lactate | 60543.14 | 2189.023 | 148.2792 | | 39946.2 | 1263.37 | 97.83425 | | 44011.24 | 3733.784 | 107.7901 | | 40830.48 | 1865.013 | |
| | | | | | | | | | | | | | | | |
| Amino acids | | | | | | | | | | | | | | | |
| Gly | 185667 | 5818 | 88 | | 211841 | 8080 | 100 | | 146168 | 1344 | 69 | | 211177 | 13877 | 100 |
| Ala | 40898 | 625 | 67 | | 51251 | 1285 | 84 | | 33272 | 545 | 55 | | 60888 | 4211 | 100 |
| Arg | 7077 | 452 | 71 | | 8236 | 770 | 83 | | 6464 | 127 | 65 | | 9902 | 442 | 100 |
| Asp | 17654 | 575 | 67 | | 19456 | 873 | 74 | | 17409 | 776 | 66 | | 26445 | 1359 | 100 |
| Asn | 9718 | 140 | 64 | | 11970 | 336 | 79 | | 8635 | 393 | 57 | | 15192 | 1516 | 100 |
| Gln | 241164 | 6395 | 72 | | 283383 | 13089 | 84 | | 197771 | 3426 | 59 | | 337154 | 9332 | 100 |
| Glu | 255679 | 8955 | 89 | | 239064 | 9387 | 83 | | 258709 | 3074 | 90 | | 287270 | 13905 | 100 |
| His | 8041 | 176 | 60 | | 10625 | 331 | 80 | | 7407 | 301 | 56 | | 13301 | 955 | 100 |
| Ile | 25843 | 330 | 72 | | 32087 | 958 | 90 | | 22531 | 446 | 63 | | 35810 | 2590 | 100 |
| Leu | 26286 | 692 | 70 | | 33730 | 899 | 90 | | 22611 | 102 | 60 | | 37506 | 2234 | 100 |
| Lys | 17365 | 951 | 70 | | 20350 | 1992 | 81 | | 15917 | 451 | 64 | | 24974 | 1171 | 100 |
| Met | 8548 | 171 | 55 | | 11717 | 545 | 76 | | 7274 | 313 | 47 | | 15489 | 646 | 100 |
| Phe | 19252 | 293 | 66 | | 24954 | 1019 | 86 | | 16943 | 381 | 58 | | 29015 | 2210 | 100 |
| Pro | 56773 | 1223 | 91 | | 61787 | 2036 | 99 | | 49904 | 548 | 80 | | 62148 | 2965 | 100 |
| Ser | 35215 | 1012 | 74 | | 42219 | 1763 | 89 | | 29061 | 1386 | 61 | | 47587 | 1763 | 100 |
| Thr | 91622 | 2003 | 65 | | 113094 | 4139 | 81 | | 73931 | 560 | 53 | | 140117 | 6702 | 100 |
| Trp | 4418 | 89 | 70 | | 6001 | 255 | 95 | | 3447 | 68 | 55 | | 6312 | 384 | 100 |
| Tyr | 19564 | 212 | 65 | | 25465 | 660 | 85 | | 17177 | 428 | 57 | | 30049 | 2044 | 100 |
| Val | 22768 | 470 | 76 | | 28292 | 888 | 95 | | 19547 | 271 | 66 | | 29763 | 1917 | 100 |
| Total | 1093552 | | 71 | | 1235525 | | 86 | | 954178 | | 62 | | 1420101 | | 100 |
| | | | | | | | | | | | | | | | |
| Glutathione metabolism | | | | | | | | | | | | | | | |
| GSH | 36927 | 13664 | 88 | | 40377 | 12406 | 97 | | 45315 | 2813 | 109 | | 41743 | 11934 | 100 |
| GSSG | 15965 | 6643 | 83 | | 17109 | 4891 | 88 | | 6905 | 1351 | 36 | | 19334 | 6698 | 100 |
| Cys-GSH | 329 | 231 | 71 | | 394 | 189 | 85 | | 27 | 55 | 6 | | 461 | 260 | 100 |
| Total | 53221 | | 86 | | 57880 | | 94 | | 52248 | | 85 | | 61538 | | 100 |
| | | | | | | | | | | | | | | | |
| ATP/GTP utilization | | | | | | | | | | | | | | | |
| ATP | 46787 | 4128 | 57 | | 46906 | 4404 | 112 | | 46778 | 876 | 112 | | 64581 | 1506 | |
| ADP | 3729 | 433 | 8 | | 3456 | 161 | 749 | | 4734 | 432 | 1026 | | 4074 | 326 | |
| AMP | 423 | 69 | 648 | | 333 | 64 | 7 | | 581 | 71 | 12 | | 425 | 44 | |
| AMP/ATP | 0.0090 | | | | 0.0071 | | | | 0.0124 | | | | 0.0066 | | |
| ADP/ATP | 0.0797 | | | | 0.0737 | | | | 0.1012 | | | | 0.0631 | | |
| | | | | | | | | | | | | | | | |
| GTP | 14106 | 1790 | 94 | | 13880 | 3074 | 93 | | 12730 | 573 | 85 | | 14959 | 1143 | |
| GDP | 776 | 103 | 115 | | 713 | 112 | 57 | | 861 | 14 | 69 | | 747 | 33 | |
| GMP | 165 | 55 | 124 | | 123 | 86 | 53 | | 198 | 22 | 85 | | 162 | 25 | |
| GMP/GTP | 0.0117 | | | | 0.0089 | | | | 0.0156 | | | | 0.0108 | | |
| GDP/GTP | 0.0550 | | | | 0.0514 | | | | 0.0676 | | | | 0.0499 | | |
| | | | | | | | | | | | | | | | |
| Others | | | | | | | | | | | | | | | |
| SAM+ | 443 | 41 | 62 | | 426 | 63 | 60 | | 324 | 11 | 45 | | 713 | 24 | 100 |
| Methionine | 297 | 40 | 149 | | 316 | 57 | 74 | | 175 | 57 | 41 | | 283 | 55 | 100 |
| 5-Oxoproli | 9507 | 221 | 108 | | 8281 | 646 | 94 | | 7801 | 877 | 89 | | 8806 | 396 | 100 |

# Supplementary Table S3

## Original data for Table 2

### 1-50

| NAME | GENE_NAME | GENE_SYMBOL | Control | IL-1β | SE | SE+IL-1β |
|---|---|---|---|---|---|---|
| A_33_P3332180 | retinol binding pr | RBP3 | 0 | 3.1276035 | 0 | 0 |
| A_33_P3402931 | FEZ family zinc fi | FEZF1 | 0 | 1.5233078 | 0 | 0 |
| A_23_P122924 | inhibin subunit b | INHBA | 0 | 1.9451256 | -0.6911979 | 0.8883085 |
| A_23_P200741 | dermatopontin | DPT | -2.38E-07 | 1.2820988 | -0.270215 | 0.05600047 |
| A_22_P00007175 | | | 0 | 1.5442185 | 0 | 0 |
| A_33_P3232334 | armadillo repeat c | ARMCX4 | 0 | 1.1324449 | 0.07354963 | 0.05951858 |
| A_33_P3272231 | major facilitator s | MFSD2A | 2.38E-07 | 2.5897312 | -0.5639885 | 1.395731 |
| A_21_P0006995 | C10orf71 antisens | C10orf71-AS1 | 0 | 1.1162629 | 0 | 0 |
| A_22_P00015476 | NCK1 divergent tr | NCK1-DT | 0 | 1.446352 | 0.25420725 | 0.16327894 |
| A_22_P00023893 | | | 0 | 3.271554 | 0 | 0 |
| A_33_P3271930 | pyrroline-5-carbo | PYCR1 | -1.19E-07 | 3.0391717 | 0.09068263 | 0.01137352 |
| A_21_P0012641 | | | 0 | 2.14086 | 0 | 0 |
| A_21_P0000091 | leucine rich repea | LRRC34 | 0 | 1.7478597 | 0.10328186 | -0.1320467 |
| A_22_P00000891 | | | -1.19E-07 | 1.8485241 | -0.0643907 | -0.0643907 |
| A_23_P150394 | FXYD domain con | FXYD6 | 0 | 2.448351 | 0.06446505 | 0 |
| A_21_P0011798 | | | 0 | 2.5645447 | 0 | 0 |
| A_23_P370454 | potassium voltage | KCNAB3 | 0 | 1.2974579 | -0.4178736 | -0.3451498 |
| A_21_P0008822 | | | 0 | 2.265798 | 0 | 0 |
| A_22_P00014394 | long intergenic nc | LINC02570 | 0 | 1.438869 | 0 | 0 |
| A_22_P00023538 | | | -2.38E-07 | 1.561491 | -0.6863155 | -0.6863155 |
| A_33_P3800664 | | | -4.77E-07 | 1.5358963 | 0.3994341 | 0.4808178 |
| A_22_P00002274 | | | 0 | 1.6643248 | 0 | 0 |
| A_22_P00000899 | | | 0 | 2.6561227 | 0 | 1.6327348 |
| A_32_P8546 | long intergenic nc | LINC00473 | 0 | 1.7972403 | 0 | 0 |
| A_22_P00015126 | lnc-SNX27-1:1 | lnc-SNX27-1 | -2.38E-07 | 3.4751182 | -0.0323826 | 0.4028206 |
| A_24_P183150 | C-X-C motif chen | CXCL3 | -1.19E-07 | 8.826342 | 0.11035371 | 7.75577 |
| A_23_P144843 | endothelial cell sp | ESM1 | -2.38E-07 | 1.6731472 | -1.3426294 | 0.09349465 |
| A_21_P0006747 | | | 0 | 2.3404965 | 0 | 0.13274217 |
| A_33_P3236247 | | | 0 | 1.2455249 | 0 | 0 |
| A_21_P0003359 | | | 0 | 1.0126529 | 0 | 0 |
| A_22_P00006075 | | | 0 | 1.9031153 | 0 | 0 |
| A_22_P00011327 | | | 0 | 1.4682894 | 0 | 0 |
| A_24_P26792 | transient receptor | TRPM6 | 0 | 3.0254712 | 0 | 0 |
| A_32_P66756 | MGAT4 family me | MGAT4D | 0 | 1.0991092 | 0 | 0 |
| A_21_P0001172 | | | 0 | 4.5763617 | 0 | 0 |
| A_32_P59302 | HIVEP zinc finger | HIVEP3 | 0 | 2.3984208 | -1.8093479 | 0.5703957 |
| A_33_P3222761 | | | 0 | 1.2456164 | 0 | 0 |
| A_21_P0010114 | uncharacterized L | LOC101929707 | 0 | 1.2657523 | 0 | 0 |
| A_23_P31073 | MYB proto-oncog | MYB | 0 | 1.8717918 | -1.2938626 | 0.33874846 |
| A_22_P00023307 | lnc-IRF2BP2-3:1 | lnc-IRF2BP2-3 | 0 | 1.6874657 | 0 | 0 |
| A_22_P00002142 | galectin-related in | GRIFIN | 0 | 1.1260366 | 0 | 0 |
| A_33_P3314081 | | | 0 | 3.2729144 | 0 | 0 |
| A_33_P3401826 | cytidine/uridine n | CMPK2 | 0 | 2.7877684 | -0.0402095 | 1.7697375 |
| A_22_P00008488 | uncharacterized L | LOC100507377 | 0 | 3.2232795 | 0 | 0.05332387 |
| A_24_P140788 | FYVE, RhoGEF ar | FGD2 | 0 | 6.0234823 | 0 | 0 |
| A_24_P215628 | defective in cullin | DCUN1D2 | 0 | 1.3303566 | 0.25113678 | 0.2821796 |
| A_33_P3380807 | toll like receptor 9 | TLR9 | 0 | 2.478219 | 0 | 0 |
| A_22_P00021003 | | | 0 | 1.6220198 | 0 | 0 |
| A_22_P00004326 | | | 0 | 1.5656009 | 0 | 0 |

### 51-100

| | | | | | | |
|---|---|---|---|---|---|---|
| A_33_P3247042 | formyl peptide rec | FPR3 | 0 | 5.161647 | 0 | 0 |
| A_24_P363408 | hes related family | HEY2 | 0 | 3.811584 | 0 | 0 |
| A_21_P0001603 | long intergenic nc | LINC01350 | 0 | 1.6429682 | -0.0975313 | 0.10699141 |
| A_33_P3343972 | radial spoke head | RSPH1 | 0 | 1.9823651 | 0 | 0 |
| A_21_P0001043 | | | 0 | 1.6570868 | 0 | 0 |
| A_23_P97141 | regulator of G pro | RGS1 | 0 | 1.2483611 | 0 | 0 |
| A_33_P3343175 | C-X-C motif chen | CXCL10 | 0 | 3.2538223 | 0 | 2.2520034 |
| A_22_P00017312 | | | 0 | 2.2732253 | 0.3285725 | 0.21969867 |
| A_33_P3400171 | AKT serine/threon | AKT2 | 0 | 2.319785 | -0.1890106 | -0.3094404 |
| A_21_P0009371 | lnc-C17orf101-1:1 | lnc-C17orf101-1 | 0 | 2.304503 | 0 | 0 |
| A_33_P3291445 | phosphatidylinosi | PIGK | 0 | 1.0593762 | 0 | 0 |
| A_24_P931736 | | | 0 | 1.2731576 | 0 | 0 |
| A_33_P3220137 | LHFPL tetraspan | LHFPL1 | 0 | 1.515471 | 0.6478869 | 0.02249467 |
| A_23_P53450 | keratin 82 | KRT82 | 0 | 4.0778685 | 0 | 0 |
| A_23_P166408 | oncostatin M | OSM | 0 | 4.2070127 | 0 | 0 |
| A_22_P00022587 | | | 0 | 1.3406134 | -0.3318901 | -0.052453 |
| A_33_P3269166 | sodium leak chan | NALCN | 0 | 1.8061662 | 0 | 0 |
| A_32_P114284 | IKAROS family zin | IKZF2 | 0 | 1.067708 | -0.1569715 | -0.1169503 |
| A_22_P00020842 | | | 0 | 2.9278007 | 0 | 0 |
| A_22_P00023799 | lnc-KCNT1-2:1 | lnc-KCNT1-2 | 0 | 1.4656801 | 0 | 0 |
| A_21_P0005516 | SEM1 26S proteas | SEM1 | 0 | 1.1427865 | 1.5603943 | 0 |
| A_21_P0004304 | lnc-CXXC5-1:2 | lnc-CXXC5-1 | -1.19E-07 | 2.90234 | -0.005445 | -0.005445 |
| A_33_P3335540 | | | 0 | 1.2563734 | 0 | 0 |
| A_22_P00014298 | serine hydrolase l | SERHL | 0 | 5.598383 | 0 | 0 |
| A_33_P3286789 | | | -1.19E-07 | 2.3022404 | -0.6059011 | -0.1714352 |
| A_33_P3288605 | | | 0 | 2.172008 | 0 | 0 |
| A_33_P6817603 | interferon regulat | IRF6 | 0 | 4.9857836 | 0.26167238 | 0 |
| A_33_P3313779 | BICD family like c | BICDL1 | 0 | 1.9546828 | 0 | 0 |
| A_23_P349398 | zygote arrest 1 | ZAR1 | 0 | 1.5478811 | 0.23339117 | 0 |
| A_22_P00012557 | SLC25A25 antiser | SLC25A25-AS1 | 0 | 2.4815674 | 0 | 0 |
| A_21_P0005073 | lnc-NOX3-3:1 | lnc-NOX3-3 | 0 | 4.0331736 | 0 | 0 |
| A_19_P00316564 | intraflagellar trans | LOC653712 | 0 | 1.0170867 | -0.13538 | -0.039396 |
| A_21_P0014615 | T helper type 2 lo | TH2LCRR | 0 | 1.7434492 | 0 | 0 |
| A_32_P456318 | serine and arginin | SRSF12 | 0 | 1.2194948 | 0 | 0 |
| A_33_P3262083 | uncharacterized L | LOC100126784 | 0 | 1.9377503 | 0 | 0 |
| A_21_P0010259 | uncharacterized L | LOC102724370 | 0 | 1.3843832 | 0 | 0 |
| A_21_P0010015 | uncharacterized L | LOC105372564 | 0 | 1.6176414 | 0 | 0 |
| A_24_P40978 | cysteine and histi | CYHR1 | -2.38E-07 | 2.9468813 | 0.11900735 | -0.2169972 |
| A_24_P135862 | | | 0 | 2.2646952 | 0 | 0 |
| A_33_P3264940 | | | 0 | 1.3170547 | 0 | 0 |
| A_33_P3287403 | | | 0 | 1.9972601 | 0 | 0.04202426 |
| A_33_P3226460 | | | 0 | 1.3239651 | 0 | 0 |
| A_33_P3286958 | beta-1,3-galactos | B3GALT2 | 1.19E-07 | 1.7978754 | -0.2475185 | 0.74950814 |
| A_23_P99625 | fat storage induci | FITM1 | 0 | 3.398786 | 0 | 0.39573908 |
| A_22_P00013650 | long intergenic nc | LINC01592 | 0 | 3.2013574 | 0 | 0 |
| A_22_P00014981 | lnc-SMC1A-2:1 | lnc-SMC1A-2 | 0 | 2.9495609 | 0.09724224 | 0.10482371 |
| A_22_P00010702 | | | 0 | 1.7728682 | 0 | 0 |
| A_21_P0002196 | long intergenic nc | LINC00486 | 0 | 1.7789464 | 0 | 0 |
| A_22_P00017338 | | | 0 | 1.1566267 | 0 | 0 |
| A_21_P0011993 | | | 0 | 4.5368867 | 0 | 2.4975734 |

101-150

# Supplementary Table S3 (continue)

151-1200

| | | | | | | |
|---|---|---|---|---|---|---|
| A_23_P397455 | activin A receptor | ACVR1C | 0 | 1.5820396 | -0.1868904 | 0.30550718 |
| A_22_P00006835 | GNG12, DIRAS3 a | GNG12-AS1 | 0 | 2.6456037 | 0 | 0 |
| A_22_P00000296 | uncharacterized L | LOC100506937 | 0 | 4.471286 | 0 | 0 |
| A_21_P0011755 | exocyst complex c | EXOC3L2 | 0 | 1.144067 | -0.0096877 | 0.01126421 |
| A_33_P3395201 | | | 0 | 1.3940258 | 0 | 0 |
| A_23_P137139 | Bruton tyrosine ki | BTK | 0 | 1.107532 | 0 | 0 |
| A_23_P75769 | membrane spann | MS4A4A | 0 | 2.4568663 | 0 | 0 |
| A_22_P00008338 | | | 0 | 1.8281674 | 1.4966199 | -0.0293221 |
| A_23_P23029 | barttin CLCNK typ | BSND | 0 | 1.6958756 | 0 | 0 |
| A_33_P3397507 | adaptor related pr | AP1S2 | 0 | 1.7328811 | 0 | 0 |
| A_23_P41470 | DExD/H-box helic | DDX60 | -1.19E-07 | 1.9244189 | -0.1136413 | 0.82127404 |
| A_21_P0005576 | lnc-AC009365.3.1 | lnc-AC009365.3.1 | 0 | 3.9771543 | 0 | 0.21867275 |
| A_23_P166779 | long intergenic no | LINC00312 | 0 | 1.0859327 | 0 | 0 |
| A_21_P0013936 | | | 0 | 1.6399417 | 0 | 0 |
| A_33_P3244808 | bestrophin 4 | BEST4 | 0 | 2.539476 | 0.4645126 | 0.74353015 |
| A_21_P0008473 | long intergenic no | LINC02296 | 0 | 1.8470025 | 0 | 0 |
| A_21_P0008353 | lnc-SMOC1-1:1 | lnc-SMOC1-1 | 0 | 1.949326 | 0 | 0 |
| A_23_P101084 | spermatogenesis | SPATA22 | 1.19E-07 | 2.1296144 | 0.03703678 | -0.2514261 |
| A_21_P0008597 | | | 0 | 3.6457944 | 0 | 0 |
| A_21_P0008357 | lnc-C14orf118-1:3 | lnc-C14orf118-1 | 0 | 1.4132011 | 0.01478124 | 0.01114535 |
| A_21_P0011645 | ANKRD40 C-term | ANKRD40CL | 0 | 2.083375 | 0 | 0 |
| A_32_P126229 | stathmin domain | STMND1 | 0 | 3.5804152 | 0 | 0 |
| A_22_P00025088 | | | 0 | 3.6357355 | 0 | 0 |
| A_24_P106542 | R-spondin 3 | RSPO3 | 0 | 2.1817002 | -0.7985663 | 0.94643855 |
| A_21_P0003706 | | | 0 | 2.1190224 | 0 | 0 |
| A_22_P00003674 | | | 0 | 1.5822754 | 0 | 0 |
| A_22_P00004642 | | | 0 | 2.5636868 | 0 | 0.5070679 |
| A_22_P00009539 | | | 0 | 1.2611217 | 0 | 0 |
| A_22_P00013310 | | | 0 | 3.3188725 | 0 | 0 |
| A_21_P0004359 | lnc-NSUN2-1:4 | lnc-NSUN2-1 | 0 | 4.139927 | 0 | 0 |
| A_24_P277367 | C-X-C motif chem | CXCL5 | 2.38E-07 | 3.0378418 | -0.30143 | 1.9903741 |
| A_33_P3313283 | cartilage intermed | CILP2 | 1.19E-07 | 1.5546885 | -0.2876301 | 0.2828815 |
| A_22_P00015619 | | | 0 | 1.7814732 | 0 | 0 |
| A_24_P38572 | nucleolar protein | NOL6 | 0 | 3.1297956 | -0.0728902 | 0.12769198 |
| A_23_P14083 | adhesion molecul | AMIGO2 | 0 | 1.3144627 | -1.1785479 | -0.128222 |
| A_22_P00018202 | | | 0 | 2.509838 | 0 | 0 |
| A_23_P425752 | tripartite motif cor | TRIM14 | 2.38E-07 | 1.5451684 | -0.7289783 | -0.0418744 |
| A_33_P3281728 | | | 0 | 2.7279797 | 0 | 0 |
| A_21_P0008360 | lnc-C14orf166B-3 | lnc-C14orf166B-3 | 0 | 2.0099964 | 0 | 0 |
| A_23_P217712 | arylsulfatase D | ARSD | -2.38E-07 | 1.8641052 | 0.00170612 | -0.1386589 |
| A_21_P0011771 | | | 0 | 2.3889365 | 0 | 0 |
| A_21_P0013399 | transmembrane p | TMEM225B | 0 | 1.4293823 | 0 | 0 |
| A_22_P00001009 | LMO7 downstrea | LMO7DN | 0 | 2.5766292 | 0 | 0 |
| A_21_P0012744 | | | 0 | 3.0567951 | 0 | 0 |
| A_23_P111402 | R-spondin 3 | RSPO3 | 0 | 3.2081766 | 0 | 1.4694798 |
| A_23_P78037 | C-C motif chemok | CCL7 | 0 | 4.1481853 | 0 | 3.1406293 |
| A_21_P0004908 | lnc-GJA10-6:1 | lnc-GJA10-6 | 0 | 1.1532321 | 0 | 0 |
| A_21_P0001638 | lnc-RP5-915N17.9 | lnc-RP5-915N17.9 | 0 | 1.2682891 | 0 | 0 |
| A_32_P55135 | SSX family memb | SSX1 | 0 | 1.5991559 | 0 | 0 |
| A_21_P0010560 | | | 0 | 2.0307255 | 0 | 0 |

| | | | | | | |
|---|---|---|---|---|---|---|
| A_24_P52887 | endonuclease, po | ENDOU | 0 | 1.4227495 | 0 | 0 |
| A_21_P0012186 | | | 0 | 1.723166 | 0 | 0 |
| A_33_P3289576 | EF-hand domain | EFHC2 | 0 | 1.4607415 | 0 | 0 |
| A_21_P0002845 | lnc-ULK4-6:1 | lnc-ULK4-6 | 0 | 1.0654287 | 0 | 0 |
| A_33_P3342830 | ATPase sarcoplas | ATP2A1 | 0 | 2.1201658 | 0 | 0 |
| A_22_P00008552 | long intergenic no | LINC00578 | 0 | 1.3313818 | 1.6987786 | 0 |
| A_33_P3208970 | zinc finger protein | ZNF683 | 0 | 1.8910165 | 0 | 0 |
| A_33_P3298057 | ATP binding cass | ABCC5 | 0 | 2.3399258 | -0.3192527 | -0.2254661 |
| A_21_P0004103 | long intergenic no | LINC02220 | 0 | 3.203382 | 0 | 0 |
| A_21_P0006643 | | | 0 | 1.067656 | 0 | 0 |
| A_23_P101972 | calpain 13 | CAPN13 | 0 | 2.1392236 | 0 | 0 |
| A_22_P00015862 | lnc-TBL1Y-3:1 | lnc-TBL1Y-3 | 0 | 1.9181013 | 0.9767754 | 0 |
| A_33_P3298612 | | | 0 | 3.1713185 | 0 | 0 |
| A_21_P0012632 | | XLOC_l2_010936 | 0 | 1.0863256 | 0 | 0 |
| A_21_P0009102 | matrix metallopep | MMP2 | 0 | 3.2324758 | 0 | 0 |
| A_33_P3237150 | bone morphogene | BMP2 | 0 | 2.2527833 | -0.5682778 | 0.7165456 |
| A_21_P0013289 | uncharacterized L | LOC646588 | 0 | 2.6685781 | 0 | 0 |
| A_33_P3216467 | family with seque | FAM83E | 0 | 1.2476592 | 0 | 0 |
| A_21_P0012512 | | | 0 | 1.4762125 | 0 | 0 |
| A_21_P0012875 | | | 0 | 3.3557835 | 0 | 0 |
| A_33_P3355694 | | | 0 | 1.2042711 | -0.0637891 | -0.0637891 |
| A_22_P00022830 | | | 0 | 3.3000712 | -0.3114657 | -0.1514397 |
| A_23_P415611 | solute carrier fam | SLC9B1 | 0 | 1.8956008 | 0.21112227 | 0 |
| A_23_P163195 | leucine rich repea | LRFN5 | 0 | 1.0803752 | 0 | 0 |
| A_21_P0002436 | | | 0 | 1.3418698 | 0 | 0 |
| A_33_P3278298 | nucleolar protein | NOM1 | 0 | 2.353109 | 0 | 0 |
| A_33_P3335177 | secreted frizzled r | SFRP4 | 0 | 4.497487 | 0.0203557 | 0 |
| A_21_P0002553 | long intergenic no | LINC01940 | 0 | 1.4232721 | 0 | 0.02044189 |
| A_21_P0005702 | | | 0 | 1.2549915 | 0 | 0 |
| A_23_P150316 | matrix metallopep | MMP12 | 0 | 6.548991 | 0 | 5.367628 |
| A_21_P0002556 | | | 0 | 2.1253114 | 2.0687792 | 0 |
| A_33_P3252588 | synaptosome ass | SNAP47 | 0 | 1.01667 | 0.31093836 | -0.0233986 |
| A_22_P00019436 | long intergenic no | LINC00681 | 0 | 1.2168651 | 0 | 0 |
| A_23_P161698 | matrix metallopep | MMP3 | 0 | 3.737667 | -0.3618302 | 2.3036466 |
| A_23_P142013 | XPA binding prote | XAB2 | -2.38E-07 | 1.1186671 | -0.0821695 | -0.0566161 |
| A_19_P00321636 | | | 0 | 3.0619755 | -0.0033777 | -0.0758157 |
| A_23_P161218 | ankyrin repeat do | ANKRD1 | -2.38E-07 | 2.3931403 | -1.0022182 | -0.1800865 |
| A_24_P259154 | tubulin alpha 3f p | TUBA3FP | -1.19E-07 | 3.5898318 | 0.38138676 | 0.2646054 |
| A_33_P3249736 | GOLGA2 pseudog | GOLGA2P11 | 0 | 3.6208773 | 0 | 0 |
| A_21_P0009510 | | | 0 | 2.2896261 | 0 | 0 |
| A_22_P00021199 | | | 0 | 1.0835676 | 0 | 0 |
| A_23_P305759 | abhydrolase doma | ABHD3 | -1.19E-07 | 2.222951 | 0.15728831 | -0.2947514 |
| A_23_P388150 | phospholipase A2 | PLA2G12B | 0 | 3.2575917 | 0 | 0 |
| A_22_P00011839 | long intergenic no | LINC02133 | 0 | 2.3359323 | 0 | 0 |
| A_22_P00016052 | gastric adenocarc | GAPLINC | 0 | 1.8521256 | -0.0463207 | -0.4719679 |
| A_22_P00022284 | | | 0 | 1.232316 | 0 | 0 |
| A_33_P3390471 | | | 0 | 1.31316 | 0 | 0 |
| A_33_P3398513 | C1GALT1 specific | C1GALT1C1L | 0 | 4.800212 | 0.24923241 | 0 |
| A_22_P00006112 | NR2F1 antisense | NR2F1-AS1 | 0 | 2.1789036 | 0.11810994 | 0.08955836 |
| A_22_P00010071 | | | 0 | 1.0609145 | -0.1239996 | -0.2139998 |

# Supplementary Table S3 (continue)

201-250

| | | | | | | |
|---|---|---|---|---|---|---|
| A_22_P00024904 | | | 0 | 3.7995205 | 0 | 0 |
| A_23_P137573 | left-right determir | LEFTY2 | 0 | 3.5210032 | 0 | 0 |
| A_23_P157865 | tenascin C | TNC | 0 | 1.6307116 | -0.9171791 | 0.53038836 |
| A_32_P173662 | cysteine rich secr | CRISP2 | 0 | 1.4778872 | 0 | 0 |
| A_32_P182394 | zinc finger protein | ZNF77 | 0 | 2.7706661 | 0 | 0 |
| A_23_P45871 | interferon induced | IFI44L | 0 | 1.6083279 | -0.5402448 | 0.3048582 |
| A_21_P0001575 | notch receptor 2 | NOTCH2 | 0 | 1.1206446 | 0 | 0 |
| A_22_P00004294 | lnc-CNOT7-1:1 | lnc-CNOT7-1 | 0 | 3.925849 | 0 | 0 |
| A_21_P0007318 | long intergenic no | LINC02709 | 2.38E-07 | 1.4937754 | -0.4437686 | -0.332647 |
| A_22_P00024919 | | | 0 | 2.569821 | 0 | 0 |
| A_21_P0003875 | | | 0 | 1.2746711 | 0 | 0 |
| A_21_P0007677 | lnc-FBXL14-2:1 | lnc-FBXL14-2 | 0 | 2.2887678 | 0 | 0 |
| A_23_P133408 | colony stimulating | CSF2 | 0 | 7.0126147 | -0.3978076 | 5.225366 |
| A_33_P3256272 | keratin associated | KRTAP10-5 | 1.19E-07 | 1.33951 | -0.2539024 | -0.0872421 |
| A_33_P3381186 | solute carrier fam | SLC5A8 | 0 | 1.896512 | 0 | 0 |
| A_33_P3331125 | solute carrier fam | SLC2A12 | -1.19E-07 | 2.7358117 | 0.6753919 | 0.01683259 |
| A_23_P124252 | calcium/calmodu | CAMK1D | 0 | 1.5358944 | 0.01474571 | -0.3443253 |
| A_23_P29965 | submaxillary glan | SMR3B | 0 | 1.0644145 | 0 | 0 |
| A_22_P00014436 | lnc-SH2B2-1:1 | lnc-SH2B2-1 | 0 | 2.5503306 | 0 | 0 |
| A_22_P00001456 | renal clear cell ca | KCCAT198 | 0 | 1.161118 | -0.0384321 | -0.0384321 |
| A_33_P3294302 | pleckstrin homolo | PHLDA1 | 0 | 2.000482 | 0 | 0 |
| A_33_P3306624 | hypocretin neurop | HCRT | 0 | 3.3569183 | 0 | 0 |
| A_22_P00022298 | | | 0 | 3.1390424 | 0 | 0 |
| A_23_P106720 | transducin beta li | TBL3 | 2.38E-07 | 1.3758554 | 0.24452448 | 0.31965637 |
| A_33_P3350580 | protein kinase C d | PRKCD | 0 | 1.6760116 | 0 | 0 |
| A_33_P3381613 | | | 0 | 2.3721313 | 0 | 0 |
| A_22_P00025561 | | | 0 | 2.618433 | 0 | 0 |
| A_33_P3354267 | akirin 1 | AKIRIN1 | -4.77E-07 | 1.4008675 | -0.1927891 | 0.17586231 |
| A_33_P3378216 | | | 0 | 2.798514 | 0 | 0 |
| A_22_P00023703 | uncharacterized L | LOC100130548 | 0 | 2.771099 | 0.15734315 | 0.29791594 |
| A_33_P3307157 | RNA binding moti | RBM20 | 0 | 1.1600108 | 0 | 0 |
| A_33_P6471837 | LBX2 antisense R | LBX2-AS1 | 0 | 2.6132822 | -0.3460164 | -0.2246199 |
| A_24_P363278 | male germ cell as | MAK | 0 | 1.3098345 | 0 | 0 |
| A_33_P3412353 | zinc finger protein | ZNF268 | 2.38E-07 | 1.4519043 | 0.29363728 | 0.10420299 |
| A_22_P00002300 | MYRF antisense | MYRF-AS1 | 0 | 3.3968463 | 0 | 0 |
| A_23_P23279 | RCSD domain con | RCSD1 | 0 | 1.7310991 | 0 | 0.60905683 |
| A_24_P140475 | sorbin and SH3 d | SORBS2 | 0 | 2.2730575 | 0 | 0 |
| A_33_P3360271 | even-skipped hor | EVX2 | 0 | 1.3808737 | 0 | 0 |
| A_23_P92410 | caspase 3 | CASP3 | 4.77E-07 | 1.0721235 | -0.70507 | -0.1511774 |
| A_21_P0010514 | macrophage stim | MST1L | -2.38E-07 | 1.2539 | -0.7203418 | -0.1288436 |
| A_22_P00017950 | long intergenic no | LINC02032 | 0 | 1.7287564 | -0.4275708 | 0.57101727 |
| A_21_P0006210 | lnc-ACTL7A-2:1 | lnc-ACTL7A-2 | 0 | 1.8020225 | 0 | 0 |
| A_33_P3407400 | sperm flagellar 2 | SPEF2 | 0 | 3.924208 | 0.28210247 | 0.0548687 |
| A_19_P00317144 | uncharacterized L | LOC101928004 | 0 | 4.920497 | 0 | 0 |
| A_33_P3292018 | | | 0 | 1.8210483 | 0 | 0 |
| A_21_P0014688 | uncharacterized L | LOC100288162 | 0 | 1.5122938 | 0 | 0 |
| A_22_P00024000 | | | 0 | 1.8140297 | 0 | 0 |
| A_23_P35148 | TATA-box binding | TAF13 | 0 | 2.5394382 | 0.12312317 | 0.36758137 |
| A_33_P3291619 | long intergenic no | LINC02649 | 0 | 1.3986902 | 0.2696954 | 0 |
| A_33_P3405459 | fibronectin type II | FNDC11 | 0 | 1.920064 | 0 | 0.36720657 |

251-300

| | | | | | | |
|---|---|---|---|---|---|---|
| A_22_P00021309 | | | 0 | 1.3178401 | 0 | 0 |
| A_33_P3286943 | | | 0 | 1.862524 | 0 | 0 |
| A_23_P363313 | solute carrier fam | SLC16A11 | -2.38E-07 | 1.830575 | -0.331492 | -0.0028231 |
| A_21_P0002880 | receptor like tyros | RYK | 0 | 1.8138824 | 0 | 0 |
| A_21_P0007656 | | | 0 | 1.0820417 | 0 | 0 |
| A_21_P0012706 | | | 0 | 2.1195483 | 0 | 0 |
| A_22_P00011747 | | | 0 | 1.7812653 | 0 | 0.3691845 |
| A_24_P813550 | | | 0 | 2.3616695 | 0 | 0 |
| A_22_P00011624 | | | 0 | 1.2896576 | 0 | 0 |
| A_22_P00018017 | zinc finger protein | ZNF192P1 | 1.19E-07 | 2.0434566 | -0.3262006 | -0.3460321 |
| A_21_P0012945 | | | 0 | 1.3715463 | 0 | 0.19372141 |
| A_23_P200138 | SLAM family mem | SLAMF8 | 0 | 2.3938136 | 0 | 1.2094924 |
| A_22_P00016876 | tumor suppressor | TUSC8 | 0 | 1.6970582 | 0.6417552 | 0.06042802 |
| A_19_P00322711 | long intergenic no | LINC00662 | 2.38E-07 | 1.4516883 | 0.1997037 | -0.1260784 |
| A_33_P3359858 | ACOXL antisense | ACOXL-AS1 | 0 | 1.7758985 | 0 | 0 |
| A_33_P3381513 | CD274 molecule | CD274 | 0 | 1.5355039 | -0.7805095 | -0.2595159 |
| A_32_P389118 | HEAT repeat cont | HEATR5B | -2.38E-07 | 1.8644447 | 0.40968513 | 0.16016626 |
| A_22_P00019233 | phospholipase C | PLCG1 | 0 | 1.4772949 | 0 | 0 |
| A_33_P3226620 | aryl hydrocarbon | AIPL1 | 0 | 4.1321177 | 0 | 0 |
| A_23_P90453 | keratinocyte diffe | KRTDAP | 0 | 7.727434 | 0 | 0 |
| A_21_P0001428 | lnc-AC092811.1-1 | lnc-AC092811.1-1 | 0 | 3.5854197 | 0 | 0 |
| A_21_P0003606 | | | 0 | 3.809752 | 0 | 0 |
| A_22_P00005118 | | | 0 | 4.9660807 | 0 | 0 |
| A_22_P00021303 | uncharacterized L | LOC101929353 | 0 | 1.7953324 | 0 | 0 |
| A_21_P0005749 | lnc-TTC35-1:1 | lnc-TTC35-1 | 0 | 3.4729648 | 0 | 0 |
| A_33_P3313221 | zinc finger protein | ZNF691 | 1.19E-07 | 1.0256867 | 0.14423466 | -0.0607185 |
| A_22_P00005350 | RXYLT1 antisens | RXYLT1-AS1 | 0 | 1.0261168 | 0 | 0 |
| A_23_P414281 | chromosome 16 o | C16orf71 | 0 | 2.2629976 | 0 | 0 |
| A_33_P3395713 | bromodomain and | BRWD1 | 0 | 1.2663312 | 0.03821349 | 0.21625209 |
| A_23_P12082 | chitinase 3 like 2 | CHI3L2 | 0 | 1.0919733 | 0 | 0 |
| A_21_P0010932 | | | 0 | 1.4191804 | 0 | 0.04433322 |
| A_23_P25155 | G protein-coupled | GPR84 | 0 | 1.2393484 | 0 | 0.11822534 |
| A_22_P00010664 | | | 0 | 3.689188 | 0 | 0 |
| A_22_P00011996 | | | 1.19E-07 | 3.0097141 | -0.1584841 | -0.2616897 |
| A_24_P244944 | multiple C2 and tr | MCTP2 | -1.19E-07 | 3.330171 | 0.53076386 | -0.1416578 |
| A_21_P0011598 | | | 2.38E-07 | 1.4110284 | 0.02897716 | -0.0445569 |
| A_22_P00025236 | | | 0 | 2.031043 | 0 | 0 |
| A_21_P0008189 | lnc-SLITRK1-2:1 | lnc-SLITRK1-2 | 0 | 1.1305032 | 0 | 0 |
| A_33_P3298539 | apolipoprotein A1 | APOA1 | 0 | 1.5963635 | 0 | 0 |
| A_22_P00006319 | | | 0 | 1.2384524 | 0 | 0 |
| A_33_P3396370 | acrosin binding p | ACRBP | 0 | 10.061849 | 0 | 0.15563631 |
| A_21_P0008985 | lnc-IRF8-2:1 | lnc-IRF8-2 | 0 | 2.122404 | 1.597723 | 0 |
| A_23_P347632 | MTSS I-BAR dom | MTSS1 | 0 | 2.223424 | -1.4306829 | 1.0668724 |
| A_22_P00013382 | | | 0 | 2.3957424 | 0 | 0 |
| A_23_P52266 | interferon induced | IFIT1 | 0 | 3.3363414 | 0.42862153 | 2.266458 |
| A_23_P87363 | ADP-ribosyltrans | ART1 | 0 | 1.0079975 | 0 | 0 |
| A_23_P76234 | rabphilin 3A | RPH3A | 0 | 1.6011596 | 0 | 0 |
| A_21_P0011230 | | | 1.19E-07 | 1.6545315 | -0.1575599 | -0.1575599 |
| A_22_P00009824 | uncharacterized L | LOC100507053 | 0 | 4.28868 | 0 | 0.02605033 |
| A_24_P302584 | SRY-box transcrip | SOX11 | 2.38E-07 | 1.4746542 | -0.1730578 | 0.37203312 |

# 301-350 Supplementary Table S3 (continue) 351-366

| | | | | | | |
|---|---|---|---|---|---|---|
| A_33_P3282522 | glucosaminyl (N-a | GCNT7 | 0 | 1.0806031 | 0 | 0 |
| A_22_P00012056 | | | 0 | 2.0998168 | 0 | 0 |
| A_23_P421306 | synaptotagmin 12 | SYT12 | 2.38E-07 | 1.2492366 | -0.6635425 | 0.15960574 |
| A_33_P3256997 | membrane spann | MS4A4E | 2.38E-07 | 3.4822721 | -0.8463545 | -0.8463545 |
| A_23_P209129 | leukocyte associa | LAIR2 | 0 | 2.088704 | 0 | 0 |
| A_23_P23669 | palmdelphin | PALMD | 0 | 1.1049037 | 0.2706263 | 0 |
| A_23_P338479 | CD274 molecule | CD274 | -2.38E-07 | 1.3761554 | -2.0044534 | -0.1164053 |
| A_22_P00007880 | | | 0 | 3.792148 | -0.2285796 | -0.1387663 |
| A_33_P3392698 | spindlin family m | SPIN4 | 0 | 4.398086 | 0.15615225 | -0.1606069 |
| A_23_P148047 | prostaglandin E re | PTGER4 | -2.38E-07 | 1.2845683 | -1.9114426 | -0.1524108 |
| A_23_P42963 | ring finger protein | RNF133 | 0 | 1.5690985 | 0 | 0 |
| A_21_P0010822 | cyclin Y like 1 pse | LOC100129055 | 0 | 3.7145424 | 0 | 0 |
| A_21_P0006386 | tRNA methyltrans | TRMO | 0 | 2.2314878 | 0.6921761 | 0 |
| A_24_P281439 | olfactory receptor | OR2T29 | 2.38E-07 | 1.3072572 | -1.2561016 | -1.2561016 |
| A_32_P48466 | lebercilin LCA5 lik | LCA5L | 0 | 2.656828 | 0.01247656 | 0 |
| A_22_P00007519 | | | 0 | 1.2280774 | 0 | 0 |
| A_22_P00025124 | | | 0 | 2.0635495 | 0 | 0 |
| A_23_P259071 | amphiregulin | AREG | -2.38E-07 | 1.1920333 | -0.9516275 | -0.9904403 |
| A_33_P3294868 | small integral mem | SMIM10L2A | -2.38E-07 | 3.4248981 | -0.1286852 | -0.0266929 |
| A_22_P00005574 | | | -2.38E-07 | 2.5579777 | -0.6878862 | -0.6878862 |
| A_33_P3232637 | | | 0 | 1.5735183 | 0 | 0 |
| A_23_P376488 | tumor necrosis fa | TNF | 0 | 1.4389868 | 0 | 0.25540555 |
| A_23_P13740 | neuron navigator 3 | NAV3 | 0 | 1.1903639 | -1.14468 | -0.0648296 |
| A_33_P3329922 | cyclin Y like 2 (ps | CCNYL2 | 0 | 1.0443854 | 0 | 0 |
| A_21_P0002459 | lnc-EXOC6B-1:1 | lnc-EXOC6B-1 | 0 | 3.3981595 | 0 | 0 |
| A_21_P0001128 | long intergenic no | LINC01389 | 0 | 1.1434801 | -0.4403064 | -0.4403064 |
| A_21_P0007904 | | | 0 | 2.0994558 | 0 | 0 |
| A_22_P00008162 | | | 0 | 1.0891533 | 0 | 0 |
| A_21_P0005726 | lnc-ZFHX4-2:1 | lnc-ZFHX4-2 | 0 | 1.982645 | 0 | 0 |
| A_21_P0003423 | | | 0 | 2.2815666 | 0 | 0 |
| A_33_P3422240 | UV stimulated sca | UVSSA | 0 | 2.1429155 | -0.089167 | 0.19522107 |
| A_22_P00007990 | | | 0 | 1.6332121 | 0 | 0 |
| A_24_P160202 | pannexin 2 | PANX2 | 0 | 2.1411052 | 0 | 0 |
| A_23_P162486 | protein tyrosine p | PTPN6 | 0 | 2.017311 | 0 | 0 |
| A_23_P23074 | interferon induced | IFI44 | 0 | 1.8140063 | 0.10369086 | 0.6943114 |
| A_22_P00017873 | | | 0 | 1.7447987 | 0 | 0 |
| A_33_P3243405 | G protein-coupled | GPR182 | -2.38E-07 | 1.8034706 | -0.2968838 | -0.3304334 |
| A_22_P00016423 | | | 0 | 1.4904513 | 0 | 0 |
| A_22_P00024290 | uncharacterized L | LOC101928441 | 0 | 1.1808386 | 0 | 0 |
| A_24_P28722 | radical S-adenosy | RSAD2 | 0 | 4.779969 | 0 | 3.7134507 |
| A_33_P3390924 | HECW1 intronic tr | HECW1-IT1 | 0 | 1.7574553 | 0 | 0 |
| A_22_P00016305 | long intergenic no | LINC02393 | 0 | 4.033458 | 0 | 0 |
| A_21_P0006250 | lnc-C9orf38-2:1 | lnc-C9orf38-2 | 0 | 1.3088932 | 0 | 0 |
| A_22_P00002855 | GRM7 antisense | GRM7-AS1 | 0 | 2.8743105 | 0 | 0 |
| A_33_P3410026 | KIAA1324 | KIAA1324 | 0 | 3.220459 | 0 | 0 |
| A_23_P12746 | mannose recepto | MRC1 | 0 | 1.926383 | 0 | 0 |
| A_23_P19816 | ring finger protein | RNF32 | 0 | 3.4830985 | 0 | 0 |
| A_33_P3321993 | | | 0 | 1.1545691 | -0.0413156 | -0.0110178 |
| A_21_P0009616 | lnc-RP11-17M16. | lnc-RP11-17M16. | 0 | 2.9239392 | 0 | 0.00373328 |
| A_22_P00003266 | | | 0 | 3.3516946 | 0 | 0 |

| | | | | | | |
|---|---|---|---|---|---|---|
| A_32_P131640 | SLIT and NTRK lik | SLITRK4 | 0 | 2.1259558 | -0.2625674 | 0.7958827 |
| A_21_P0012221 | ankyrin repeat do | ANKRD20A11P | 0 | 1.3896141 | 0 | 0 |
| A_21_P0005831 | lnc-OPRK1-1:1 | lnc-OPRK1-1 | 0 | 2.1335883 | 0 | 0 |
| A_33_P3395028 | long intergenic no | LINC02085 | 0 | 1.2162719 | -0.065971 | 0.13407505 |
| A_32_P137966 | DEP domain cont | DEPDC4 | 0 | 2.32025 | 0 | 0 |
| A_24_P185158 | reticulophagy regu | RETREG3 | 2.38E-07 | 1.9740667 | 0.2215085 | 0.10964775 |
| A_33_P3399748 | SECIS binding pro | SECISBP2 | 0 | 2.2340546 | 0 | 0 |
| A_22_P00011306 | | | 0 | 3.2400904 | 0 | 0 |
| A_22_P00016433 | uncharacterized L | LOC107105282 | 0 | 2.9366093 | 0 | 0 |
| A_33_P3297005 | essential meiotic | EME2 | 0 | 1.2459345 | 0 | 0 |
| A_21_P0007693 | lnc-IFLTD1-1:1 | lnc-IFLTD1-1 | 0 | 1.2429194 | 1.395464 | 0 |
| A_24_P156748 | solute carrier fam | SLC30A2 | 0 | 2.6195254 | 0 | 0.20121181 |
| A_24_P140608 | heparin binding E | HBEGF | 2.38E-07 | 1.3451309 | -2.9231434 | -0.9620426 |
| A_22_P00001871 | | | 0 | 2.1243901 | 0 | 0 |
| A_22_P00004345 | | | 0 | 4.925886 | 0.20313537 | 0.23461425 |
| A_33_P3327723 | | | 1.19E-07 | 1.9180155 | 2.3621402 | -0.1726456 |
| A_22_P00022314 | mutS homolog 6 | MSH6 | 0 | 2.3813586 | 0 | 0 |

# Supplementary Table S4

## Intracellular and extracellular concentration of *p*-coumaric acid

| | *p*-Coumaric acid | | | | | |
|---|---|---|---|---|---|---|
| | Intracellular (amol/cell) | | | Extracellular (μM) | | |
| SE+IL-1β | 511 | ± | 64 | 139 | ± | 7 |
| SE | 332 | ± | 80 | 146 | ± | 15 |
| IL-1β | 0 | ± | 0 | 0 | ± | 0 |
| Control | 0 | ± | 0 | 0 | ± | 0 |
